# Visualizing the internalization and biological impact of nanoplastics in live intestinal organoids by Fluorescence Lifetime Imaging Microscopy (FLIM)

**DOI:** 10.1038/s41377-025-01949-0

**Published:** 2025-08-12

**Authors:** Irina A. Okkelman, Hang Zhou, Sergey M. Borisov, Angela C. Debruyne, Austin E. Y. T. Lefebvre, Marcelo Leomil Zoccoler, Linglong Chen, Bert Devriendt, Ruslan I. Dmitriev

**Affiliations:** 1 Tissue Engineering and Biomaterials Group, Department of Human Structure and Repair, Faculty of Medicine and Health Sciences, Ghent University, The Core, C. Heymanslaan 10, 9000 Ghent, Belgium; 2 Ghent Light Microscopy Core, Ghent University, 9000 Ghent, Belgium; 3 Institute of Analytical Chemistry and Food Chemistry, Graz University of Technology, Stremayrgasse 9, Graz, 8010 Austria; 4 Calico Life Sciences LLC, South San Francisco, CA 94080 USA; 5 Bio-Image Analysis Technology Development Group, DFG Cluster of Excellence “Physics of Life”, TU Dresden, 01307 Dresden, Germany; 6 Laboratory of Immunology, Department of Translational Physiology, Infectiology and Public Health, Faculty of Veterinary Medicine, Ghent University, 9820 Merelbeke, Belgium

**Keywords:** Biophotonics, Imaging and sensing, Nanoparticles

## Abstract

Increased micro- and nanoplastic (MNP) pollution poses significant health risks, yet the mechanisms of their accumulation and effects on absorptive tissues remain poorly understood. Addressing this knowledge gap requires tractable models coupled to dynamic live cell imaging methods, enabling multi-parameter single cell analysis. We report a new method combining adult stem cell-derived small intestinal organoid cultures with live fluorescence lifetime imaging microscopy (FLIM) to study MNP interactions with gut epithelium. To facilitate this, we optimized live imaging of porcine and mouse small intestinal organoids with an ‘apical-out’ topology. Subsequently, we produced a set of pristine MNPs based on PMMA and PS (<200 nm, doped with deep-red fluorescent dye) and evaluated their interaction with organoids displaying controlled epithelial polarity. We found that nanoparticles interacted differently with apical and basal membranes of the organoids and showed a species-specific pattern of cellular uptake. Using a phasor analysis approach, we demonstrate improved sensitivity of FLIM over conventional intensity-based microscopy. The resulting ‘fluorescence lifetime barcoding’ enabled distinguishing of different types of MNP and their interaction sites within organoids. Finally, we studied short (1 day)- and long (3 day)-term exposure effects of PMMA and PS-based MNPs on mitochondrial function, total cell energy budget and epithelial inflammation. We found that even pristine MNPs could disrupt chemokine production and mitochondrial membrane potential in intestinal epithelial cells. The presented FLIM approach will advance the study of MNP toxicity, their biological impacts on gastrointestinal tissue and enable the tracing of other fluorescent nanoparticles in live organoid and 3D ex vivo systems.

## Abstract (web summary)

Fluorescence Lifetime Imaging Microscopy (FLIM) combined with pristine model NIR MNPs visualizes the internalization and biological impact of the nanoplastics in live small intestinal organoids

## Graphical abstract

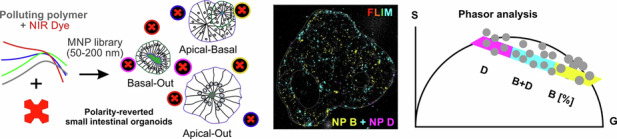

## Introduction

Plastics pervade every aspect of modern life, from packaging and household goods to applications in agriculture and medicine. Their durability, versatility and other attractive features have driven their widespread adoption but simultaneously contribute to their resistance to (bio)degradation. As a result, the use of slowly degrading polymers in everyday plastic products has resulted in the global accumulation of micro- and nanoplastics (MNPs) across diverse ecosystems, including soils, aquatic environments, the atmosphere, and even food sources like meat, drinking water, and vegetables. These MNPs interact with the biosphere in complex ways, giving rise to an emergent ecological niche termed the ‘plastisphere'^1–3^. Despite growing interdisciplinary efforts to mitigate plastic pollution through environmental remediation and the development of degradable polymers, the plastisphere is poised to remain a global societal challenge for decades to come.

Humans and animals are mainly exposed to MNPs via inhalation and ingestion. Disturbingly, MNPs have already been detected in human blood, the brain, the gut, the reproductive system, and various other organs^4–7^. While precise estimates of MNP intake in livestock and other animal species remain unavailable, exposure levels are likely comparable. The presence of MNP in both humans and animals raises pressing questions about their potential health effects. Limited studies in rodents and human cell lines have shown that MNPs can negatively impact the intestinal epithelium, disrupting cell metabolism as well as triggering oxidative stress and inflammation in the gut^8–10^. However, it remains unclear whether these effects occur in human tissues or across diverse animal species in a physiologically relevant three-dimensional context. Real-world plastic particles can display a complex size and shape distribution and can be associated with contaminating metals, bio- and living matter traces. However, to establish a fundamental understanding of MNP uptake mechanisms and their effects on tissues, ‘model’, weathered or ‘pristine’ particles are needed^1,11^.

To address the complexity of the MNP interactions with tissues, the use of tractable 3D tissue models is essential. Advances in induced pluripotent stem cell and adult stem cell technologies now enable the recapitulation of key aspects of tissue development and environmental interactions using organoid models. For instance, ‘mini-guts’ or intestinal organoids closely mimic the cellular composition, functionality, 3D architecture, and extracellular matrix organization of the native intestinal epithelium. Thereby providing a robust platform to investigate MNP interaction with the gut epithelium and help to develop multi-organ on-a-chip models^12–15^.

While organoid models are highly amenable to ‘omics’ and bulk analysis methodologies, their true complexity can be grasped best with imaging methods such as live cell fluorescence microscopy, full-field OCT and related approaches^14,16,17^, which provide multiparametric, quantitative readouts, together with long-term dynamic imaging. Fluorescence lifetime imaging microscopy (FLIM) presents an additional advanced method, allowing multiparametric and multi-dimensional 5D imaging (X,Y,Z, *t* and luminescence lifetime (tau, τ) dimensions)^17,18^. FLIM enables the study of intra- and intercellular processes using biomarkers and a growing list of physical-chemical parameters pertinent to cell function, including inflammation and cell metabolism^17^. The latter is tightly connected with cell growth, differentiation, “live vs. death” decisions and the stem cell niche environment^19^, all of which can be replicated in 3D organoid models^14^. In addition to sensing and measuring cellular physiology in live organoids, FLIM can also be used to trace MNPs. The latter can exhibit unique and material-specific autofluorescence or can be loaded with various fluorescent dyes^17^. However, in respect to MNP, FLIM-based approaches focused so far only on the analysis of purified and separately present MNP in relatively simple sample solutions^20–22^. Such ‘intrinsic’ spectral properties and ‘mixing with the dye’ approaches can be challenged by the strong autofluorescence in live organoids^23,24^ and provide limited information on the MNP type, localization and composition.

To address the need for a more accurate analysis of the structure-activity relationships of MNP interaction with tissues, we report a live-cell FLIM approach, using optimized small intestinal organoid cultures and deep red dibutoxy-aza-BODIPY dye-impregnated pristine nanoparticles for studying MNP interactions with the gut epithelium. We demonstrate that our approach enables efficient lifetime-based separation of different polymers and highly sensitive MNP detection. This approach can be combined with long-term dynamic imaging of MNP internalization sites and their effects on cellular energy production mechanisms and inflammatory responses.

## Results

### Design, spectral and fluorescence lifetime characterization of pristine nanoparticles mimicking MNPs

**Fig. 1:**
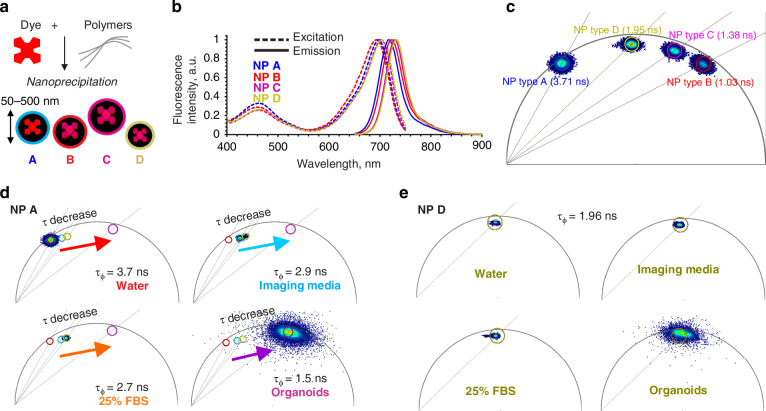
Design, spectral and fluorescence lifetime characteristics of the model nanoplastics NP A-D. **a** Schematics of NP design. **b** Normalized fluorescence excitation and emission spectra of NP A-D. **c** Phasor-FLIM characterization of NP A-D (8 months after production; 0.5 mg/ mL, deionized water, 37 ^o^C). Fluorescence decays were collected with 80 MHz frequency of laser pulse. Centre τ_ϕ_ values indicated in brackets. **d, e** Effect of the environment on the phasor plots of NP A (**d**) and NP D (**e**), respectively. Nanoparticles were measured in all solutions at 0.5 mg/mL, with exception of organoids (10 µg/mL, incubated for 18-24 h)

Various types of polymers can be used to produce dye-doped nanoparticles via nanoprecipitation and achieve controlled size and distribution^25–27^. Considering the need in live imaging, photostability, hydrophobicity and organoid live cell autofluorescence, we selected dibutoxy-aza-BODIPY dye (diBuO-aza-BODIPY^28^, exc. 700 nm/em. 720 nm) as an emitter for doping the polymeric particles. For the ‘pristine’ polymer MNP and proof-of-principle testing, we selected negatively and positively charged polystyrene (PS) and poly(methylmethacrylate) (PMMA)-based structures^25,29^. Both PMMA and PS are abundant types of MNP^30^ and are widely used in bio- and nanosensing applications across the life science domains^31–33^. Using a nanoprecipitation method, we produced 4 types of deep red emitting nanoparticles (NP A-D) with a comparable size and dispersity, matching characteristics of the previously described MMIR1 nanosensor^26^ (100 ~ 200 nm, based on dynamic light scattering, and 58 ~ 134 nm, based on TEM, Fig. 1, s1 and Table 1). To address the presence of different charged groups in MNPs and improve their water dispersibility, we used quaternary amine-modified PMMA (RL100), methacrylic acid (MA)- and polyvinylpyrrolidone (PVP)- modified PMMA and PS polymers. As expected, we found high brightness and deep red fluorescence (exc. 694 ~ 703 nm with em. max. 717 ~ 732 nm, depending on the polymer) with all 4 NP types (Fig. 1b, Table 1). We also estimated the fluorescence quantum yield of the dye in polymeric particles. Compared to the solution in chloroform (Φ = 36%)^34^ the brightness is decreased significantly in RL-100, PS-MA and PS-PVP (Φ = 10%, 18%, and 12%, respectively) and drastically in PMMA-MA (Φ = 4%). Although this is a very rough estimation due to significant scattering in PS-PVP and PS-MA particles, the data suggests some degree of aggregation and quenching of the chromophore in the particles. Despite that the brightness is sufficient for the measurements in all the cases, the data suggests plenty of room for further improvements by using dyes with brighter emission (higher Φ) and better compatibility with the polymeric materials.

**Table 1:** Properties of dibutoxy-aza-BODIPY-doped nanoparticles

NP type (polymer)	I_exc_, nm	I _em_, nm	Z_av_, nm (DLS)	zeta-potential, mV (DLS)	Z_av_, nm (TEM)	τ, ns (Fluo)	τ_φ_ in water (‘FLIM’, phasor), ns	Intensity weighted τ _m_ in water, ns (FLIM, decay fitting)	χ^2^
A (RL100)	698	717	n.d.	n.d	78	4.4	3.71	4.02	2.447
B (PMMA-MA)	694	723	99	−29	81	1.1	1.03	1.15	0.938
C (PS-PVP)	703	729	200	−7	99	1.5	1.38	1.53	1.145
D (PS-MA)	703	732	166	−28	135	2.3	1.95	2.21	1.216

Z_av_ values obtained via dynamic light scattering (nanoparticles in deionized water) and TEM are presented. τ values were measured on fluorescence spectrometer (‘Fluo’), τ_ϕ_ and intensity-weighed τ_m_ values were obtained on FLIM microscope (‘FLIM’) via phasor (80 MHz) or decay fitting approaches (see Methods for more details)

Interestingly, depending on the polymer and the presence of the charged groups, the fluorescence lifetimes (analyzed by three individual methods, see Table 1, Fig. 1c, S2) in water for all the 4 types of the NPs showed a broad range from 1 to 4 ns with the multi-exponential fluorescence decays. The fluorescence lifetime of the dye depends on the chemical structure of the polymer and observed differences could originate, for example, from partial aggregation of the dye in the matrix or the sensitivity of the luminescent properties to different environmental parameters^26,35^. The non-encapsulated dye did not display longest lifetime value (~2.2 ns in DMSO, Figure S2). For NP A, consisting mostly of PMMA and acrylate units bearing quaternary ammonium groups (RL-100), we observed a strong influence of the environment on the fluorescence lifetime (roughly estimated by τ_ϕ_ phasor lifetimes), generally decreasing from 3.7 in deionized water to 2.7 and 1.5 ns (Fig. 1d) in presence of FBS and in contact with live organoids, respectively. In contrast, NP D (Fig. 1e), that are based on copolymer of styrene and maleic acid, showed generally constant fluorescence lifetime: τ_ϕ_ = 1.96 ns in water, imaging medium, 25% FBS or when taken up by organoids. Such striking differences in fluorescence lifetimes, based on the type of the polymer structure and charge represent an attractive ‘barcoding’ feature to distinguish between different types of MNP within the same spectral (excitation-emission) channel. Importantly, τ_ϕ_ and τ values observed on different detection platforms matched well, confirming reliability of the phasor FLIM measurements (Table 1). We also observed no changes in observed lifetimes upon 20+ months of storage, confirming their stability (Figure S2). We subsequently illustrated this ‘barcoding’ feature by fusing drops of NP A and B solutions (NPs with the longest and the shortest fluorescence lifetime in water) and performing phasor analysis of the FLIM data (Figure S2). The resulting phasor density cluster, with G and S coordinates plotted from fluorescence decays of corresponding FLIM image pixels, displayed a typical indicative elongated shape and was aligned between the positions of the two distinct NP A and NP B emitting species clusters (Figure S2a-b). According to the phasor principle, the distance of the fused cluster points from one of the distinct species clusters reflects the weight of this species in a mixture at the corresponding pixel location on the image^36^. Thus, the analysis of fused phasor cluster point coordinates can be applied for detecting the multiple emitting species with spatial resolution, if individual fluorescent species lifetimes are not strongly affected by the local environment. With the assumption that fluorescence lifetime is an indicative parameter of distinct emitting species (including system noise), a simple detection of the phasor clusters with the corresponding G and S coordinates can be used to detect the potentially rare events of NP uptake by tissues.

To estimate the reliability of the dibutoxy-aza-BODIPY-labeled NP cluster position on a phasor plot in comparison to the system noise signal (which includes optical noise, short noise or thermal noise), we reconstructed phasor clusters for TCSPC-FLIM measurements of different dilutions of NP D in water (0.58-500 µg/mL) and compared them to the signal from water containing no NPs, which can be referred as a “system noise” sample (Figure S3). The dilution of homogeneous NP D solution led to a decrease in fluorescence intensity and accordingly to a decrease in the photon rate per pixel. This decrease in the pixel photon count upon ~1000-fold dilution (from total photons count 9.9*10^7^ for 500 μg/mL solution decay to 1.4*10^5^ photons for 0.58 μg/mL solution decay measured for 512 × 512 pixel resolution image, binning 1) correlated with the increase in NP D phasor cluster points dispersion from the theoretical center of the cluster, indicated as the center of the circular ROI mask (Figure S3, magenta). However, the principal position of the NP D phasor cluster on a plot remained intact within the same ROI mask (magenta ROI, Figure S3). Importantly, we could narrow down the scattering by applying the pixel binning and improving the photon counts per pixel. At the same time, we did not observe any random localization of the noise cluster points on a phasor plot within the area of the NP D phasor ROI. In contrast, pixel binning reduced noise pixels scattering by clearly segregating their localization in a short lifetime zone of the phasor plot (yellow ROI). Thus, even with a considerable scattering of the points from the main cluster position, the dibutoxy-aza-BODIPY-labeled NP cluster could be reliably detected with the pixel binning approach, improving the event detection reliability. Remarkably, 0.58 μg/mL NP D concentration in water still was not a limit for the detection of the NP D cluster on a plot without shifting its general position towards the noise and corresponding changes of the τ_φ._ The presented data is in contrast to a similar approach^20^ utilizing TCSPC-based phasor plot analysis of NP in water, where due to low signal-to-noise ratio the achieved limit of detection of non-stained polystyrene nanoparticles was only 10 μg/mL. This difference in sensitivity might be due to a different microscopy instrument set up as well as the NP labeling with a near-infrared aza-BODIPY dye, which is characterized by a fairly high quantum yield and allows to keep a high signal-to-noise ratio upon dilution of the NP.

### Visualization of apical-out organoid polarity reversion via live microscopy

**Fig. 2:**
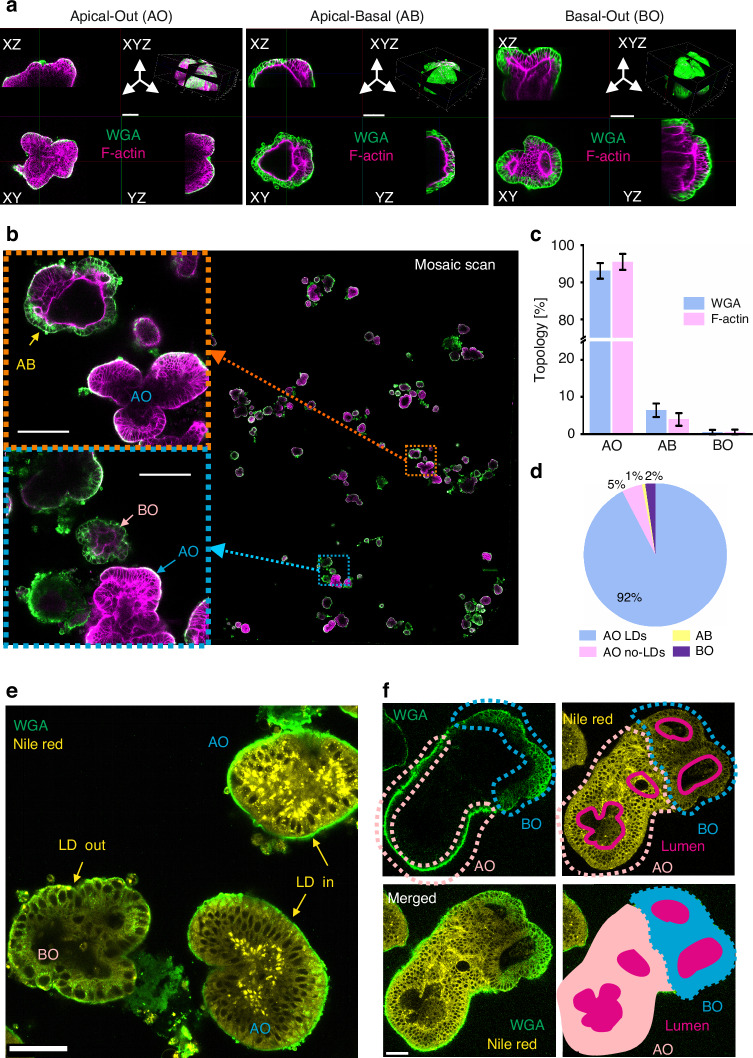
Fluorescent Wheat Germ Agglutinin (WGA) conjugate and Nile Red enable validation of the apical-out topology in live small intestinal organoids. **a** Representative 3D confocal images of WGA-Alexa Fluor 488 and phalloidin–Texas Red (F-actin labeling) co-stained organoids with apical-out (AO), apical-basal (AB) and basal-out (BO) topology. The scale bar is 100 μm. **b** Large field mosaic scan of PFA-fixed pig intestinal organoids after 18 h of polarity reversion, co-stained with fluorescent WGA and phalloidin. *Left:* representative images of AO, AB and BO organoids, indicated on mosaic image (*right*). The scale bar is 100 μm. **c** Analysis of the AO, ABO and BO topology confirms a major population of polarity reverted organoids, estimated with WGA staining and F-actin labeling, respectively. Histogram plot shows average percentage of WGA/F-actin topology types counted from 4 independent mosaic scan experiments (see table S2). **d** Topology analysis based on WGA and Nile Red labeling for polarity reverted organoids, quantified from the mosaic scan experiment: AO with lipid droplets (AO LDs) and AO without lipid droplets (AO no-LDs), AB and BO. **e** Lipid droplets display a characteristic distribution in AO organoids, contrasting with BO. **f** Co-staining with WGA and Nile Red reveals organoid structure in apical-basal organoid (AB). The scale bar is 50 μm

An ‘apical-in’ or Basal-Out (referred as BO in the text) topology of small intestinal organoids represents a major limitation of this model for studying direct interactions of the apical membrane with nutrients, microbiota and pathogens. While this has been addressed by microinjection or partial disaggregation to crypts and re-assembly^14^, a more elegant approach was introduced by the group of Amieva^37,38^ for human enteroids, via depletion of ECM and subsequent transition to suspension culture. We subsequently adapted this protocol to the cultures of porcine and mouse small intestinal organoids and confirmed the phenomenon of the polarity reversion, using staining of F-actin as a conventional marker of the apical membrane^38^ (Figure S4). Since the process of polarity reversion can differ from organoid to organoid, requiring live monitoring of organoids topology, we evaluated two tracers enabling labeling of an Apical-Out (AO) topology in live small intestinal organoids: fluorescent wheat germ agglutinin (WGA) and Nile Red, labeling the cell membrane and lipid droplets (LD), respectively (Figure S5, Fig. 2). Both tracers display attractive spectral and fluorescence lifetime properties (~2.1 ns for WGA-Alexa Fluor 488 and ~3.3 ns for yellow-red emission of the Nile Red (Figure S5B, S5C, S5E, Table 2).

**Table 2:** Fluorescent probes used in live microscopy of organoids

Probe	Physiological parameter	Stock concentration (Solvent)	Staining concentration	Excitation/Emission (nm)	Fluorescence Lifetime (τ, ns), [citation]
AF488-WGA	Binds N-acetyl-D-glucosamine and sialic acid; mucous membranes^72^	1 mg/mL (PBS)	4 μg/mL	490/495 - 520	1.2-2.6 ns [this study]
Fluorescein-UAE-1	Glycoproteins, glycolipids with α-fucose residues^73^	5 mg/mL (PBS)	5 μg/mL	490/495 - 520	NA (No staining observed)
Nile Red	Solvatochromic dye. Lipids, LDs, cytoplasm.	1 mM (DMSO)	2 μM	490/588 - 620	2-4 ns^68^
Mito Tracker Green (MTG)	Mitochondria	10 μM (DMSO)	100 nM	490/497 - 551	0.5-1.3 ns [this study]
Mito Tracker Red (MTR)	Mitochondria	10 μM (DMSO)	100 nM	581/594 - 654	0.8-1.2 ns [this study]
TMRM	Mitochondrial polarization	10 μM (DMSO)	10 nM	540/559 - 585	1.5-2.5 ns^52^
Phalloidin-Texas Red	F-actin labeling in fixed cells	66 μM	0.132 μM	591/608 - 625	3-3.3 ns [this study]
Phalloidin-Alexa Fluor 546	F-actin labeling in fixed cells	66 μM	0.132 μM	556/566 - 585	Intensity
LysoSensor Green DND-189	Lysosomes	1 mM (DMSO)	2 μM	448/471 - 535	Intensity
IgG conjugated with Alexa Fluor Plus 488	Secondary antibody	2 mg /mL	2 μg / mL	488/494 - 542	Intensity

Fluorescence lifetime was measured using FLIM microscope (see ‘Microscopy’)

*WGA* wheat germ agglutinin, *UAE-1* *Ulex europeaus* agglutinin-1, *TMRM* tetramethylrhodamine methyl ester

To evaluate WGA labeling of the organoids, we first performed an analysis of the small intestinal organoid topology after 18 h suspension culture (Table S1) and found 3 topological states: Apical-Out (AO, mainly outer membrane labeling with WGA, Figure S5a and S5g), Basal-Out (BO, deeper cellular borders labeling with WGA and internalization of WGA, Figure S5d and S5g) and Apical-Basal (AB, both AO and BO characteristic staining visible in different parts of the organoid) (Figure S5g). We then validated organoid topologies with co-localization experiments using live WGA staining followed by fixation and F-actin staining (Fig. 2a). To quantify the total number of each type, we performed mosaic imaging of organoids co-stained with WGA and F-actin (Fig. 2b) and found a similar percentage of AO (WGA: 93.1%, F-actin: 95.5% and BO (WGA: 0.53%, F-actin: 0.55%) topology in organoids with both labelling approaches (Fig. 2c).

To test if lipid droplet distribution is a reliable marker for AO identification, we classified organoids to AO, AB and BO by WGA labeling and then quantified lipid droplets in AO organoids via mosaic imaging (Figure S5h). We found that most AO organoids contained LDs (AO with LDs: 92%, AO without LDs: 5%), confirming Nile Red-labeled LDs as a reliable identifier for AO morphology (Fig. 2d). Thus, we concluded that Nile Red provides similar topological information on organoids through the spatial distribution of lipid droplets (LDs), localized at the basal membrane side^39^, i.e., within the internal region of AO and external region of BO organoids (Fig. 2e). We also observed that the Nile Red signal was higher in AO compared to BO organoids, while WGA staining intensity remained similar between these two organoid types (Figure S5D, S5E). Statistical analysis within a defined AO region of interest (ROI) and BO ROI from mosaic scanned images using FIJI (Figure S5f, S5h), confirmed Nile Red intensity being a reliable marker for discriminating between AO and BO organoids (*p* = 0.006).

Collectively, combining Nile Red with WGA-Alexa Fluor 488 provided better information on the organoid topology in live organoid imaging. Importantly, Nile Red is compatible only with live microscopy as it does not remain in organoids after fixation, in contrast to WGA labeling. An advantage of Nile Red and WGA co-staining is in more defined classification of the internal organoid structure, allowing identification of lumen (for BO structures) and pseudo-lumen regions (for AO structures) within the live organoids (Fig. 2f). This is in contrast with many other fluorescent tracers, such as UEA-1, live actin tracking probes (e.g. CellMask Actin Tracking Stains and SPY-actins, data not shown) and mitochondrial stains (MTG, TMRM and MTR), which demonstrated negative or differential staining within the population of organoids in suspension or parts of the individual organoids (Figure S5I). This phenomenon can be explained by a potential presence of mucus in the AO organoids or a preferential uptake of the dyes through basal or apical membranes of the gut organoids, which has to be further elucidated.

### Nanoplastics display specific accumulation in small intestinal organoids

**Fig. 3:**
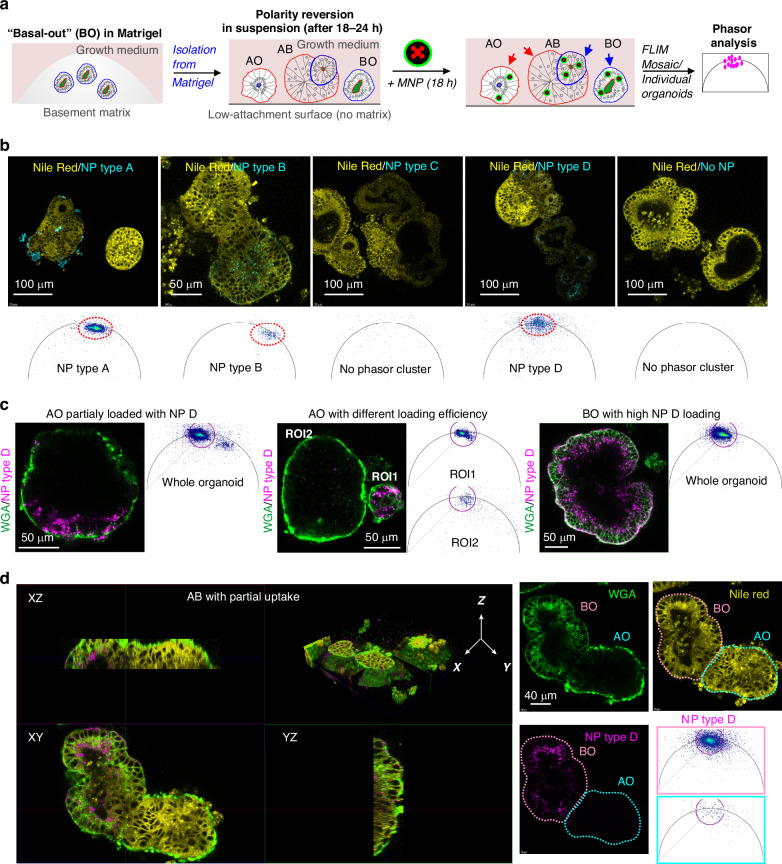
MNPs display diverse staining and uptake in the small intestinal organoids. **a** Scheme of experimental workflow. **b** Representative confocal fluorescence images and corresponding phasor plots of the pig small intestinal organoid incubated with NP A-D (10 μg/ mL, 18 h), co-stained with Nile Red. NP A, B and D displayed signals on both fluorescent intensity images and phasor plots, whereas type C showed no signal, similar to control. **c** Different types of uptake of NP type D uptake (10 μg/mL NPD, 20% laser power) into organoids, co-stained with WGA, with respect to the topology and size. *Left:* partial NP D uptake in AO, showing high pixel signal on a phasor plot. *Middle:* organoid size-dependent NP D uptake in AO. ROI 1: enhanced NP D pixel signal in a small AO, ROI 2: reduced NP D pixel signal in larger organoid. *Right:* Homogeneous NP D distribution in BO with strong signal on a phasor plot. **d** Topology-dependent uptake of NP D (magenta) in apical-basal organoid (AB), co-stained with WGA (green) and Nile Red (yellow). *Left:* 3D reconstruction shows distinct NP D distribution in AO and BO regions. *Right:* representative fluorescence image of AB organoid taken from 3D reconstruction. AO and BO regions defined by WGA and Nile Red signals, with corresponding NP type D phasor FLIM plots of these regions (bottom right). Phasor plots correspond to individual plane images

Having optimized polarity reversion and live FLIM methodology, we could now expose live organoids to the 4 types of designed NP (A-D) (Table 1, Fig. 3a). We used a ‘standard’ incubation time (18–24 h) and staining concentration (10 μg/mL), as frequently employed in staining of cells and 3D cultures with fluorescent and phosphorescent nanosensors^24,26,27,35,40–42^. Strikingly, with these conditions, we observed differences in the interaction of NP with porcine intestinal organoids: NP C (PS-PVP) showed no uptake, NP A (PMMA with quaternary ammonium groups, RL100) mostly accumulated at the organoid membrane, while both negatively charged NP types B (PMMA-MA) and D (PS-MA) showed specific intracellular uptake (Fig. 3b). Phasor plots also revealed an appearance of recognizable clusters within the specific lifetime distribution zones (see Fig. 1c) for NP A, B and D after NP exposure, while NP C and control (no NP) had no detectable signal (Fig. 3b). Thus, NP A, B and D demonstrated interaction with and internalization by intestinal epithelial cells, independently of the backbone structure (PMMA or PS) and the presence of charged groups (quaternary ammonium or methacrylate groups). This pattern was consistent across 3 different small intestinal organoid lines (porgJ-2, Fig. 3; porgJ-3 and porgJ-4, also seen in NPs localization in porgJ-2 organoids at higher magnification, Figure S6B) developed from 3 animals, suggesting animal-independent general uptake of these NPs by the pig intestinal epithelium.

Following WGA labeling, we found that NP D uptake depended on the organoid topology: thus AO organoids exhibited both high (ROI1, Fig. 3c middle panel) and low efficiency loading patterns (ROI2, Fig. 3c middle panel), in some cases belonging to the same organoid body (AO organoid with partial loading, Fig. 3c left). At first glance most AO organoids displayed lower NP D internalization in contrast to BO organoids (Fig. 3c, left panel) or BO zones in AB organoids, showing high efficiency and uniform uptake of NP D. This was confirmed by the live 3D reconstruction of AB organoid co-labeled with WGA and Nile Red (Fig. 3d). Differences in uptake from either apical or basal membranes imply potentially different routes of MNP delivery into the cells in vivo: either from the lumen of the gastrointestinal tract (e.g. with food or water) or from the bloodstream, i.e. with the injections. To confirm that organoids displayed a barrier function and the presence of tight junctions, we performed immunofluorescence of NP B and NP D-loaded organoids with anti-ZO1 antibody (Figure S7). Since the internalized vesicles with NPs showed dynamic intracellular mobility and partial co-localisation with LysoSensor Green (Figure S8, supplementary videos S1, S2), we can conclude that at least in part, their uptake is mediated by endocytosis.

We reasoned that such differences in interaction with cell membranes, caused by the type of MNP and the composition of the cell membrane, could result in species-specific differences in MNP uptake. To test this, we produced AO intestinal organoids from Lgr5-GFP reporter mice^43^ (Figure S9). Interestingly, BO and AB mouse organoids were rarely observed in the analyzed organoid population. Thus, we focused only on the interaction of NPs with AO organoids. The AO topology was confirmed by assessing the lipid droplet staining distribution with Nile Red staining and the shifted position of nuclei towards the basal membrane in polarity-reverted organoids^37^ (Figure S9). In addition to A, B and D types of NP, we also detected rare events of NP type C accumulation in mouse AO organoids (Figure S9C). For NP C, two different loading patterns were found: (i) low intensity zones (magenta mask, Figure S9) within the organoid cells with lifetime ~1.35 ns (similar to the fingerprint lifetime of NP C in water, Fig. 1c) and bright particles in rare cells with a lifetime ~1.95 ns (red mask, Figure S9D). This contrasted to the control group of unloaded organoids, where even the combined phasor plot from 8 individual microscopy images did not show appearance of the similar phasor clusters (Figure S9e). Overall, all tested NP types tended to accumulate in specific cell zones (GFP-negative differentiated cells and ‘low intensity GFP’ early differentiated daughter cells) in organoids, with almost no accumulation within Lgr5-GFP^high^ intestinal epithelial stem cells. The exception was found only for NP A (Figure S9a), where ROI-based phasor plot analysis revealed accumulation inside the stem cell zones. In contrast to a predominantly surface membrane clustering of NP A in pig organoids, NP A accumulated only inside the cells within the mouse organoids. Collectively, these data highlight the importance of species-specific effects of MNP interaction with absorptive intestinal epithelium and prompt for further investigation using controlled cell and mucus composition and the growth medium, which can affect cell metabolism and senescence^44,45^.

Finally, we studied the rate of MNP uptake in pig jejunum organoids by focusing on NP type D (Figure S10). Using a confocal microscopy with mosaic scanning we continuously monitored organoids for 24 h after addition of NP D. Strikingly, NP D adhered to the membrane of some individual organoids already after 30 min (Figure S10A). Subsequently, the MNP displayed slow internalization with rare particles detected inside organoids after 5 h of incubation (Figure S10B) with almost complete uptake (low signal on the organoid surface) after 24 h (Figure S10C). This suggests that in real-life situations, MNP may require a short exposure in order to ‘stick’ to the epithelial cells, while their physiological effect can occur with delay, caused by cell-specific internalization and accumulation.

### Phasor FLIM event counting approach reflects the number of MNP internalization events

**Fig. 4:**
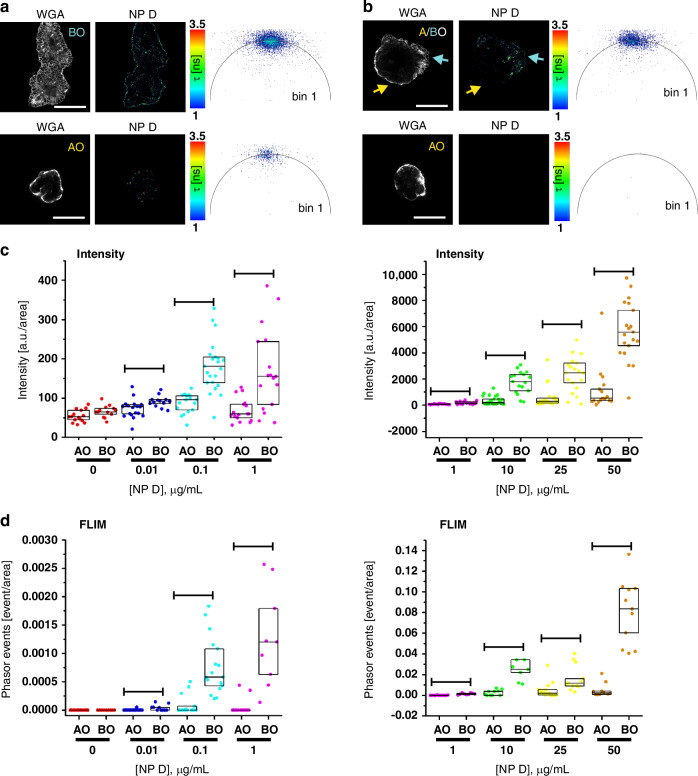
Phasor FLIM event counting approach estimates MNP uptake in organoids with improved reliability over a broad concentration range in comparison to intensity-based detection. **a**, **b** Representative images of NP D uptake (10 µg/mL, 18 h) in pig intestinal organoids in respect to their apical-basal topology with high loading in both AO and BO (A) and high loading in BO and zero at AO (B). Scale bar is 50 µm. **c, d** Comparison of NP D uptake in AO vs. BO organoids as a function of loading concentration (0-1 µg/mL, left panel and 1-50 µg/mL, right panel) on a widefield fluorescence microscope (intensity-based approach, C), and confocal FLIM microscope (phasor FLIM event counting approach, D). FLIM events were counted from the phasor plots reconstructed in the napari phasor plugin from the exported list of G and S coordinates. Results of one of the two independent experimental replicates are shown. Both C and D data were produced from the same samples. The box charts represent 25, median and 75 percentiles with dots corresponding to individual intensity or event count values normalized by ROI area values. Statistical comparison between AO and BO groups over a range of loading concentrations was performed by Mann-Whitney test (lines represent detected statistical difference at significance level *p* < 0.05). For more detailed analysis see tables S3, S5, S6 and Materials and Methods section. Statistical analysis of another independent experimental repeat is presented in tables S4, S7, S8

We next wondered if our FLIM barcoding approach could help quantifying MNP uptake by the organoids. As mentioned above (Fig. 3), AO organoids displayed generally lower nanoparticle accumulation than BO organoids. We therefore decided to have a closer look on NP D accumulation in the pig polarity reverted organoids in respect of their apical-basal topology. To increase the number of MNP-loaded organoids we performed incubation with NP D during the polarity reversion process. Indeed, we observed a similar loading tendency in the organoids, where NP D internalization in BO or AB organoids was the highest with particles concentrating at the basal membrane and some localized at the apical membrane (Fig. 4a, b). In contrast to BO organoids, which always displayed NP D uptake, AO organoids showed two distinct patterns: (i) organoids with MNP localized in vesicle-like NP bodies inside cells (Fig. 4a bottom panel) and (ii) organoids displaying no MNP accumulation (Fig. 4b bottom panel). NP D accumulation sites (referred as NP ‘cellular bodies’) inside organoids in all positive cases was clearly recognizable by the appearance of characteristic NP D phasor clusters on the phasor plots reconstructed from the organoid ROI. We noticed that the number of phasor cluster points (referred as ‘phasor FLIM events’) closely reflected the amount of fluorescent NP D ‘cellular bodies’ in the organoids. Knowing that every plotted phasor point corresponds to a pixel in the FLIM image with a certain photon count data, we hypothesized that simple point counting in phasor clusters from the characteristic lifetime zone would reflect the total number of NP ‘cellular bodies’ inside organoids. Thus, we evaluated fluorescence lifetime events detection to count internalized MNP.

We also tested if the pixel binning could improve phasor clustering for NP accumulated in organoids, similar to the solution-based experiments with NP (Figures S2, S3). To have a more dispersed phasor cluster of NP D, we decreased the NP concentration to 1 µg/mL and performed imaging of non-loaded and NP D-loaded BO organoids with different excitation power (10 and 30%), keeping all other imaging acquisition parameters unchanged. Phasor plots were reconstructed from the imaging data sets by variation of pixel binning (1, 3 and 6) and phasor thresholds (1 and 4) in LAS X software. No random points or clusters were detected in the lifetime zone of τ_ϕ_ ~ 2-1.96 ns (Figure S11, magenta circular ROI) on any of the reconstructed phasor plots of control organoid image when varying the excitation intensity. Application of a threshold parameter reduced the amount of noise cluster points, while pixel binning resulted in clustering of points in a short lifetime zone within corresponding circular ROI (Figure S11, yellow ROI). In contrast, a dispersed cluster of points appeared on the phasor plots (bin 1) of the NP-loaded organoids. The number of points increased by changing the excitation intensity to 30%. Some cluster points had a distinct drift toward the “noise” ROI, which was mitigated by exclusion of the noise affected pixels with the threshold application. Application of pixel binning further decreased the dispersion, while keeping the cluster position within the same lifetime zone. The minor shift of the cluster toward the longer lifetime zone could be explained by the improved photon statistics of individual pixels, making the phasor points position less sensitive to noise during the calculation of the G and S coordinates. Overall, implementing pixel binning and setting thresholds, in addition to median filter-based point grouping enabled a robust reconstruction of phasor clusters. Thus, we confirmed the advantage of pixel binning^46^, efficiently improving a signal-to-noise ratio and making detection of a one-point event more reliable in case of MNP.

Next, we compared the phasor FLIM event counting approach with conventional intensity-based fluorescence measurement of NP D uptake in organoids over a range of loading concentrations of 0-100 µg/mL. Examples of reconstructed phasor plots of organoids loaded with different NP D concentrations are shown in Figure S12. First, we organized intensity and phasor FLIM events data based on the organoid topology and NP loading concentrations and compared values of AO and BO organoids in each ‘concentration’ group. Both intensity and event number values were higher in BO organoids as compared to AO organoids across all loading concentrations (0.01-100 μg/mL), except for the control group (Fig. 4c, d; Table S3, 4). This result supports our observation on preferential basal membrane route of uptake of NP D (Fig. 4a) and confirms that both analytical approaches similarly reflect NP uptake in organoids.

Seeing that AO and BO organoids demonstrated a completely different NP D accumulation patterns, we reorganized the data as AO and BO groups to analyze the effect of loading concentration on the intensity and phasor FLIM events. Kruskal-Wallis ANOVA comparison of NP D-stained organoids showed a statistical difference (at p level 0.05) of both intensity and FLIM signal events between groups with the similar tendency of median parameter values, proportional to the concentration of nanoparticles (Tables S5-S8). Importantly, we did not detect phasor FLIM events in ‘no NP’ control organoids (40 organoids analyzed in total in both experimental replicates R1 and R2) with the chosen analysis settings, which made a rare event count value more significant (Figure S12, Fig. 4c, d; Tables S3-4). In contrast, the intensity values of the ‘no NP’ control organoids (both AO and BO) overlapped with the intensity values of the low concentration groups (0.01–1 μg/mL; Fig. 4c, left panel). Based on the ‘reliability’ of one event detection, we assumed that phasor FLIM can be used to count rare events of MNP accumulation in organoids. Thus, we counted the probability of organoid loading with NP D (a percentage of organoids with at least one detected phasor FLIM event from a total number of imaged organoids) as a function of NP D concentration in the surrounding media and found that the uptake per organoid increased with the concentration of NP D (Tables S3-S4). However, BO organoids were able to reach almost 100% loading probability at lower NP D concentrations (~0.1 μg/mL) than AO organoids (~10 μg/mL). This suggests the existence of intrinsic mechanisms ‘protecting’ the intestinal epithelium from NP D uptake from the apical membrane. At the same time, the heterogeneity in loading efficiency among AO organoids advocates the involvement of other factors, e.g. cell composition or metabolic state, facilitating NP uptake from the apical side. From a global perspective, the described event-based detection and analysis of heterogeneous NP uptake in the polarity-reverted enteroid culture can be viewed as an advanced approach with a potential to reflect a complex and perhaps competitive interactions of MNPs with the intestinal tissue.

### Phasor FLIM method enables analysis of complex mixtures of MNP in organoids

Real-world MNPs often display strong (poly)dispersity and a complex chemical composition, differing not only in the type of polymer backbone but also in their (partial) modifications and adsorbed ‘non-polymer’ components^47^. In addition, human and animal tissues can be exposed to multiple types of MNP at the same time. We therefore attempted to determine if our phasor FLIM event counting approach could discriminate between different nanoparticle types within the same organoid. Pristine dye-doped nanoparticles displayed a predictable and ‘calibrated’ fluorescence lifetime linear trajectory on their phasor plots (Fig. 1), while the phasor FLIM event counting reliably reflected the number of internalized nanoparticles (Fig. 4, S12). Thus, our goal was to utilize both properties to quantitatively trace a mixture of NP with distinct fluorescence lifetime values (e.g. NP B and NP D) in organoids.

**Fig. 5:**
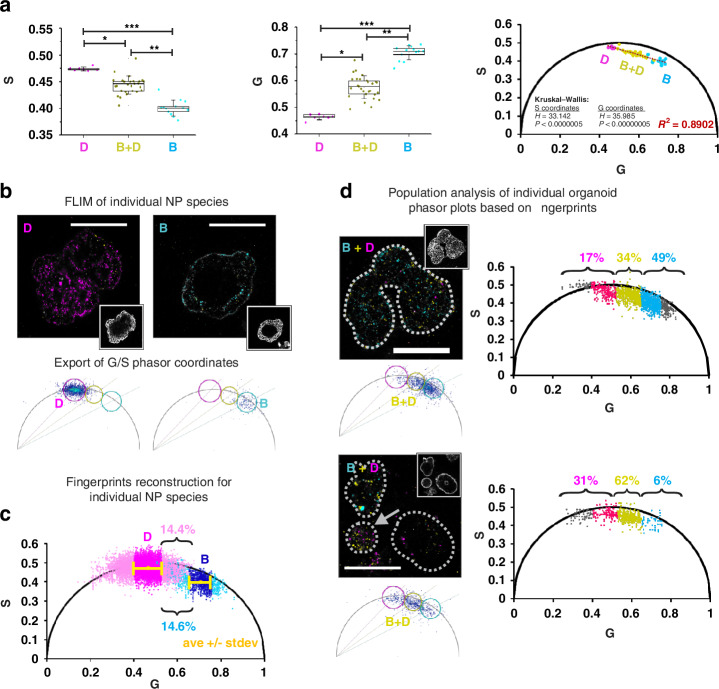
Phasor FLIM event counting approach resolves heterogeneous MNP populations in intestinal organoids. **a** Distinct positions of internalized NP D, NP B and B/D mixture clusters on a phasor plot. From left to right: comparison of average S and G coordinates of phasor cluster groups and their position on a phasor plot. Box plots represent 25 and 75 percentiles, while whiskers show standard deviation. Each point corresponds to average G or S values of individual organoids. N_D_ = 7, N_B_ = 10, N_DB_ = 28. Asterisks indicate statistical difference between groups detected with Kruskal-Wallis ANOVA and following Dunn’s post-hoc test (*p < 0.05; **p < 0.0005; ***p < 0.0000005). **b**–**d** Illustration of the main steps of the fingerprint phasor FLIM event counting approach applied for tracking of heterogeneous MNP in organoids. **b** Typical examples of NP D and NP B loaded BO organoids with corresponding phasor plots reconstructed in LAS X software. **c** Phasor plot with fingerprint clusters of NP D (light magenta) and NP B (cyan) in organoids reconstructed in Excel from exported G and S coordinates. Yellow color indicates average cluster coordinates, magenta and dark blue color indicate the range of G_ave_ ± SD. **d** Analysis of NP uptake from the mixture of NP D and NP B in individual organoids with apical-basal topology. *Right:* phasor plots of individual organoids, reconstructed in Excel. The numbers show the percentage of total cluster points in NP D, D/B and B lifetime zones. Magenta and cyan color indicate cluster points in the range of corresponding G_ave_ +/- SD; yellow indicates D/B zone. All MNP intensity images have pseudo color masks (magenta, yellow and cyan) based on the circular ROIs of the LAS X-produced phasor plots (NP D zone with τ_ϕ_ = 2.1 ns in magenta, intermediate D/B zone with τ_ϕ_ = 1.5 ns in yellow, and NP B zone with τ_ϕ_ = 1.0 ns in cyan, circle radius range is 46-36). Grayscale (bottom right) inserts show WGA labeling in the corresponding organoids. The scale bar is 100 µm

First, we confirmed that internalized nanoparticles composed of pure NP species and their mixture could be clearly distinguished on a phasor plot. We compared the average G and S coordinates of phasor cluster points obtained from organoids loaded with 10 μg/mL NP D, NP B and their 1:1 mixture and detected a significant difference between their values, allowing us to claim that these coordinates could be used as a marker of composition for the internalized nanoparticles (Fig. 5a). Plotting of the representative cluster points with averaged coordinates demonstrated the presence of three lifetime zones on a phasor plot corresponding to pure D and B nanoparticle species and the intermediate ‘B + D’ zone, similarly to solution experiments (Figure S2). However, a real phasor cluster consists of many points reflecting a NP uptake event and is potentially affected by the local NP environment and noise. Pure NP species cluster points deviate from the ‘center of a cluster’ within a distinct lifetime range (Fig. 5b), while mixed NP cluster points can spread across all three lifetime zones, reflecting the proportion of the pure NP species presented simultaneously at the same pixel of the organoid image (Fig. 5d). To count the percentage of intermediate cluster points in different lifetime zones of the phasor plot, we propose to apply a ‘lifetime fingerprint’ concept to determine range of the zones. Figure 5b–d demonstrates the step-by-step implementation of this concept for analysis of organoids loaded with the mixture of NP. The fingerprint clusters of pure D and B species were reconstructed from a sum of corresponding individual organoid clusters (7-10 per each group), allowing an increase in the number of events per phasor fingerprint and providing better characterization of their heterogeneity. For simplicity, we chose only G coordinates as a representative of τ_ϕ_ values to set fingerprint zones for NP D and NP B. Accordingly, an average G ± standard deviation (SD) value was calculated as 0.4646 ± 0.0628 for NP D and 0.7056 ± 0.0524 for NP B and used as a threshold to determine the range of the intermediate zone (Fig. 5c). Thus, zone ranges were set as: G ≤ 0.5275 for NP D, G ≥ 0.6532 for NP B and 0.5275 < G < 0.6532 for the intermediate zone (B + D). The percentage of fingerprint cluster points detected in the intermediate zone did not exceed 15% from the total amount of the individual NP types, indicating that most of the fingerprint points were in the range of a corresponding lifetime zones (Fig. 5c). Using G coordinate ranges we classified and calculated the percentage of points from ‘B + D’ clusters (Figure S13, Table S9). Examples of individual organoid ‘fingerprint’ phasor plot clusters with presented point classification analysis are shown in Fig. 5d. The false color mask applied to the intensity image of internalized nanoparticles corresponds to the selected ROIs on the phasor plots and reflects the composition of the nanoparticles. Interestingly, organoids differed in their accumulation of NP D or B types, as evidenced by different cluster point percentages in the pure D and B lifetime fingerprint zones (Table S9). Some organoids (e.g. B_D_12_2, B_D_12_3 and B_D_16) demonstrated a high percentage of points (>50%) in the ‘B + D’ zone, related to accumulation of different NP types in the same vesicles or endosomes, probably due to utilization of the same internalization mechanism^48,49^. However, some organoids accumulated mainly one type of NP (e.g. organoids B_D_12_1, B_D_22 and B_D_14_2), as evidenced by cluster point percentages reaching up to 70-80% in some fingerprint zones. These results indicate the existence of at least 2-3 individual pathways of NP D and B internalization leading to accumulation of NPs in different vesicles and/ or reflects the difference in uptake related to cell composition of the organoids. We compared accumulation of the different NP types between AO and BO organoids and did not detect differences (Figure S13) within each lifetime zone between AO and BO organoids, however, we observed a tendency among BO organoids to accumulate NP D (*p* = 0.085, U = 50 vs. critical U value 45).

In summary, we confirmed that the fingerprint cluster classification approach can be used for the analysis of intestinal organoids exposed to a complex mixture of MNPs, including the internalization pathways and possible synergism of nanoplastics during their uptake.

### Short-term exposure with nanoplastic particles does not affect cell energy production, mitochondrial function and inflammatory response

Lastly, we looked at how the potential physiological effects of the MNP could be assessed within our experimental settings. Within the intestinal epithelium and organoids, MNP can affect a multitude of processes, including the functions of the stem cell niche, absorptive and secretory cells, cell signaling and host-pathogen interactions^50^. We chose NP D (PS-MA) for these analyses, as they showed efficient accumulation in pig small intestinal organoids from the basal and apical sides.

**Fig. 6:**
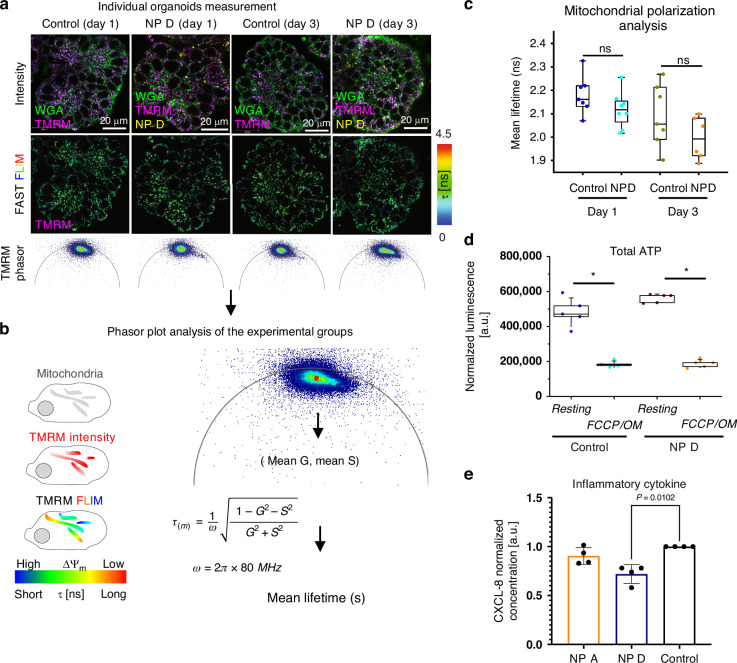
Assessment of physiological impact of internalized NP D on mitochondrial polarization, cell energy budget and inflammatory cytokine expression. **a**–**c** Workflow to investigate temporal effect of NP exposure on the mitochondrial membrane potential (ΔΨ_m_) in the organoids. **a** Representative intensity images of BO organoids co-stained with WGA and TMRM, together with fast-FLIM images and phasor plots for TMRM, after 1- and 3-days NP D exposure. **b** The scheme illustrates measurement of ΔΨ_m_ with TMRM FLIM (left) and quantification algorithm for the organoid TMRM mean lifetime (right). **c** TMRM-based mitochondria polarization analysis in control and NP D-treated BO organoids (day1: N_cont_ = 7, N_NPD_ = 8; day3: N_cont_ = 7, N_NPD_ = 6; Mann-Whitney test, significance level *p* < 0.05). Each point on a box plot represents TMRM τ_m_ for each individual organoid. Box plots correspond to 25 and 75 percentiles, whiskers correspond to 10 and 90 percentiles. **d** Effect of NP D exposure on total cellular ATP in organoids. OM-FCCP indicates organoids pre-treated with 7 μM oligomycin, 1.4 μM FCCP. No significant difference was observed between groups at resting (Rest) and under O/F stimulation. In comparison, ATP levels were significantly different between Rest and O/F conditions inside each individual organoid group, reflecting organoid response to F/O disruption of ATP production. Box plots correspond to 25 and 75 percentiles, whiskers correspond to standard deviation, *N* = 5. * - *p* < 0.05. **e** ELISA analysis of CXCL-8 expression in organoids after exposure to NP D (48 h). Data represent the mean ± SD of *N* = 4 biological independent animals with 2 repeat experiments, analyzed with Kruskal-Wallis test

Energy production is of paramount importance for any living cell. In eukaryotes, mitochondria play a vital role in this process. To determine whether NPs can affect mitochondrial homeostasis, we employed tetramethylrhodamine methyl ester (TMRM), a standard mitochondrial membrane potential probe^51^, to visualize mitochondrial polarization via FLIM^17,52^. When used in the intensity mode, TMRM only labels active mitochondria, however, this can be complemented by FLIM, visualizing lower mitochondrial polarization with longer lifetimes and more active mitochondria with shorter lifetimes^52^ (Fig. 6). We cultured organoids with and without NP D for 1 and 3 days, followed by WGA and TMRM labeling and FLIM. Intensity images confirmed WGA and TMRM labeling in the control organoids, and merged signal of WGA, TMRM and NP D in the NP treated organoids (video S3 and S4). Fast-FLIM images capturing TMRM lifetime distribution across all experimental conditions, with corresponding phasor plots for each group are presented in Fig. 6a. To look deeper in the effect of NP D on the fluorescence lifetimes of TMRM, we extracted pixel information (G, S coordinates) from phasor plots using the napari-FLIM-phasor-plotter (video S5), calculated mean G and S coordinates for each organoid and converted them into mean TMRM lifetime (Fig. 6b, right panel). While we found no significant differences in the TMRM lifetimes between the experimental groups over a 3 day period, we observed a downward trend in lifetime (Fig. 6c). While the reduced value in lifetime was small (NP D group: 2.075 ± 0.073 ns; control group: 2.236 ± 0.102 ns; Δ = 0.16 ns), it could indicate an increase in mitochondrial membrane polarization in organoids after 3 days of NP D treatment (Figure S14a). It should be noted that different cell types in organoids are expected to display differences in cell cycle and mitochondrial activity^53,54^. It is therefore important to perform cell-specific segmentation for different cell types for such an analysis, to account for subtle changes of cell-specific metabolism upon MNP exposure.

Since mitochondria are mobile organells^55,56^, we also looked at the applicability of the live mitochondrial tracking tool Nellie for our experimental setup (Figure S15a). However, dynamic analysis of linear and angular velocity, length, and mitochondrial area did not show reproducible trends in three replicates (Figure S15b-c). For instance, while the first replicate displayed reduced mitochondrial area in NP D-treated organoids compared to controls at day 3, the second replicate showed no difference and the third replicate showed increased mitochondrial area in NP D-treated organoids. Such inconsistencies can be a result of technical limitations of the used imaging approach (*XYt* scanning confocal microscopy with ‘fast FLIM’), not grasping mitochondrial dynamics in 3 geometrical dimensions (i.e. *XYZt*). Indeed, we observed rapid mitochondrial movement in our organoid model (video S6) with an estimated linear velocity of 0.02 ~ 0.03 μm/s. At our optimized image acquisition settings (1024×1024 pixels, 400 Hz), each single-plane image acquisition required 2.59 s, while multi-sequential Z-stack of 5 depth coordinates would require 13 s for one image sequence, being too slow for tracking mitochondria in 4D. Potential future studies and development of live light-sheet FLIM-compatible systems would help addressing this issue, at least with the presented model^18,56–58^. Additionally, our mitochondrial tracking approach did not account for such aspects of functional complexity of apical-out organoids, as the cellular composition and the different cell cycle stages.

With no clear trend observed for the effect of NP D on mitochondrial polarization and mobility, we measured total ATP in NP D-treated and control organoids (Fig. 6d, S14b). While we forced the OxPhos to increase by using a low glucose medium^53,59,60^, we did not observe significant changes in ATP at resting and under mitochondrial uncoupling conditions, upon oligomycin and FCCP treatment. Taken together, these results also confirm that the selected ‘pristine’ NP at the chosen range of incubation conditions, does not affect overall mitochondrial mobility and cell energy budget in the majority of cells in the gut organoids. However, our analysis did not account for cell-type specific effects, which would be possible by adding live imaging tracers, such as markers of enterocytes, stem cells and others, and cell-specific segmentation^61^.

Intestinal epithelial cells not only provide a barrier function, but also communicate with underlying immune cells. Even in a steady state, the gut epithelium secretes chemokines and cytokines to attract and inform immune cells. For instance, CXCL-8, a chemokine involved in neutrophil migration, is constitutively secreted by intestinal epithelial cells and is increased in response to pathogens or their virulence factors^62,63^. To determine whether exposure of gut organoids to NP influenced the communication network of the gut epithelium, we assessed CXCL-8 secretion levels in control and NP A and D treated organoids. As shown in Fig. 6e, type D nanoparticles seemed to decrease CXCL-8 secretion levels by gut organoids. This can potentially result in reduced neutrophil numbers in the gut tissues and ultimately to an impaired ability to clear enteric infections.

Collectively, we illustrate here that the presented experimental model is compatible with functional assessment using live FLIM, dynamic intracellular organelle tracking and ‘bulk’ downstream assays, such as ELISA and ATP luminescent assays. This opens possibilities for a range of additional multi-parameter readouts, such as fluorescent cell sorting, single cell sequencing, mass spectrometry imaging, multiplexed immunofluorescence and others.

## Discussion

Here, we developed a new approach, based on the use of bright NIR pristine nanoparticles, optimized dynamic imaging of live polarity-reverted small intestinal organoids and improved phasor FLIM-based inter-organoid tracing, interactions with different plasma membrane domains and quantification. This methodology enables deeper understanding of the structure-activity relationships of MNP interaction with tractable in vitro and potentially multi-organ-on-a-chip and in vivo live tissue models. The presented ‘lifetime barcoding’ feature allows for detecting different types of MNP and potentially their interactions with a non-plastic matter, e.g. ions, biomolecules or metals. The potential limitation of presented approach can be in the need of designing ‘pristine’ nano- and microparticles for each tested type of MNP but in principle this can be also applied to real-world MNPs, pre-labeled with dyes, such as Nile Red or dibutoxy-aza-BODIPY, presented here. Importantly, the accurate choice of the labeling dye can benefit sensitivity, reliability and a ‘bar coding’ resolution of the proposed FLIM event counting approach, stemming from high quantum yields, NIR spectral properties and a well-characterized fluorescence lifetime response to environment. Another potential limitation lies in the organoid model itself, with somehow limited lifespan (so only the short-term interactions of MNPs can be analysed) and lack of additional tissue and cell types, i.e. microbiota, neurons, muscle cells etc. However, these can be potentially addressed by the use of organoid-on-chip and tissue on-a-chip models.

We have also illustrated application of methodology for assessing physiological effects on intestinal organoids, using dynamic live confocal and FLIM microscopies and downstream assays, such as ATP analysis and ELISA, meaning that it can be compatible with flow cytometry, -omics, multiple immunofluorescence and other downstream assays.

Remarkably, even with the limited selection of tested MNP, we found species-specific accumulation (different between mouse and pig) and minor effects on cytokine secretion and mitochondrial polarization. This emphasizes the importance of single cell dynamic live microscopy imaging and specific cell type segmentation approaches, over the ‘bulk population’ assays, which can mask the cell-specific effects of MNP on the living cells.

## Materials and methods

### Polymers

Composition and properties of the polymers, used to produce nanoparticles (A-D) Eudragit RL100 (NP A), PMMA-MA (NP B), PS-PVP (NP C) and PS-MA (NP D) were reported previously^25,29,64,65^.

### Small intestinal organoid culture materials

Lipidure^TM^-CM5206 (AMS.52000034GB1G, Amsbio, UK), Matrigel growth factor-reduced (734-0269, VWR, Belgium), human intestinal organoid growth medium (STEMCELL technologies, 06010, Belgium), mouse intestinal organoid growth medium (STEMCELL technologies, 06005, Belgium), DMEM high glucose GlutaMax^TM^ Supplement media (61965026, Gibco, Belgium), 0.5 M EDTA solution (15575020, Invitrogen, Belgium), 24-well Tissue Culture plates (734-2325, VWR, Belgium), sodium valproate (P4543, Sigma-Aldrich,Ireland), CHIR99021 (SML1046, Sigma-Aldrich,Ireland), PBS (18912-014, Gibco, Belgium).

Microscopy multi-well dishes were assembled using cover glass and silicon multi-well frame (cover glass with no. 1.5 thickness, e.g. μ-slide 12-well, Ibidi GmbH, Germany, or equivalent). Imaging was performed in phenol red-free DMEM (Sigma-Aldrich, D5030), supplemented with 10 mM glucose (G8270, Sigma-Aldrich), 1 mM pyruvate (11360-070, Gibco, Belgium), 2 mM GlutaMAX^TM^ (35050-038, Gibco, Belgium) and 10 mM HEPES-Na pH 7.2 buffer (15630-080, Gibco, Belgium) referred as imaging media (IM).

### Fluorescent probes

Alexa Fluor 488-conjugated Wheat Germ Agglutinin (WGA; W11261, Invitrogen, Belgium), Fluorescein-labeled *Ulex europaeus* Agglutinin I (UEA I; VEC.FL-1061-5, Vector Laboratories, Belgium), Nile Red (72485-100MG, Sigma-Aldrich, Belgium), MitoTracker Green (M7514, Invitrogen, Belgium), LysoSensor Green (DND-189, Invitrogen, Belgium), MitoTracker Red (M22425, Invitrogen, Belgium), Tetramethylrhodamine, methyl ester (TMRM) (T668, Invitrogen, Belgium), phalloidin-Alexa Fluor 546 (Invitrogen, A22283), Texas Red-X phalloidin (T7471, Invitrogen, Belgium), anti-ZO1 mouse monoclonal primary antibody (clone ZO1-1A12, 33-9100, Invitrogen, Belgium). The details of used working concentrations are listed in Table 2.

### Production and characterization of pristine nanoplastics (NP)

Eudragit RL100, PMMA-MA, and PS-MA nanoparticles were obtained via nanoprecipitation according to previously published procedures^25,29,64,65^. Briefly, dibutoxy-aza-BODIPY dye^28^ and the polymer were dissolved in a water-mixable organic solvent, water was added rapidly under stirring and the organic solvent (along with most of water) was removed under vacuum. We used tetrahydrofuran:acetone (1:3 v/v) to dissolve PS-MA, tetrahydrofuran:acetone (1:9 v/v) for PMMA-MA (polymer first dissolved in tetrahydrofuran and the solution diluted with acetone) and acetone for RL100. The concentration of the polymers in the organic solvents was 0.004 wt%. in all cases. We used 0.75 wt% of the dye in respect to the polymer for the production of NPs. After removing of organic solvents, NP dispersions were concentrated to ∼1.5-2 mg /mL of the polymer content.

PS-PVP beads are commercially available and were stained via swelling in tetrahydrofuran:water mixtures as reported previously^29^. PS-PVP beads were doped with 0.75 wt% of the dye.

The size distribution and morphology of the nanoplastics were assessed using transmission electron microscopy (TEM) as described previously^26^. Briefly, NP were drop cast (2 µl, 20 µg/ml) and dried overnight at room temperature (RT) on Formvar/Carbon-coated hexagonal copper mesh grids (FCF200H-CU-TB, Electron Microscopy Sciences). Nanoplastics (*n* = 150) were observed on a transmission electron microscope JEM 1010 (Jeol, Ltd, Japan) equipped with a charge-coupled device side-mounted Veleta camera (Emsis, Germany). Nanoparticle size was measured manually using ImageJ software (NIH, USA) and graphs were made using Graph Pad Prism 9.

The fluorescence excitation and emission spectra were measured on a Fluorolog-3 luminescence spectrometer (Horiba, Germany) equipped with a NIR-sensitive R2658 photomultiplier (Hamamatsu, Germany) in a quartz cuvette as described previously^26^. The fluorescence lifetimes of NP in water, fetal bovine serum (FBS) and other solutions were measured on a Stellaris 8 Falcon FLIM microscope (see section ‘Microscopy’ below).

Relative fluorescence quantum yields in THF were estimated according to Crosby and Demas^66^ using a solution of the same dye in chloroform (φ = 36%)^34^, excited at 650 nm. In case of PS-PVP and PS-MA NPs, subtraction of the light scattering background was performed in the range 560-765 nm.

### Pig and mouse small intestinal organoid cultures

Pig small intestinal organoids were produced from the jejunum (Table S10) as described previously^62^. Organoid lines used in the experiments were developed from 3 individual animals. Three organoid lines (porgj2, porgj3, porgj4) were used for studying MNP interactions with the intestinal epithelium. The lines porgj2, porgj3, pigD, pig13 and pig24 were used for assessing effects on chemokine expression. The porgj2 line was used for assessing total ATP, mitochondrial polarization and mitochondrial mobility. Validation of phasor plot-based counting method and MNP co-loading analysis were done on porgj3 line. Mouse small intestinal Lgr5-GFP organoid line (Lgr5-EGFP-ires-CreERT2)^43^ was provided by J. Puschhof and H. Clevers (Hubrecht Institute, Utrecht University, The Netherlands). For the detailed information on organoid lines see Table S10.

Mouse and pig organoids were cultured at density 150–300 organoids per 50 μL Matrigel dome in 500 μL of organoid growth medium per well of 24-well plate. Human IntestiCult organoid growth medium HGM (STEMCELL technologies, 06010, Belgium) was used for culturing pig organoids^62,67^. Mouse IntestiCult organoid growth medium MGM (STEMCELL technologies, 06005, Belgium) supplemented in-house with 1 mM sodium valproate and 3 μM CHIR99021 was used for Lgr5-GFP mouse intestinal organoids^53^. To prevent accumulation of dead cells in the organoid lumen and growing large structures, organoids were passaged by mechanical disruption of Matrigel domes, as described previously^68^. Resulting suspension was diluted to 10 mL with washing medium (DMEM high glucose GlutaMax^TM^ Supplement, Gibco, 61965026, Belgium), collected by centrifugation (300 g, 5 min, 4 ˚C), the supernatant was gently aspirated and the pellet was resuspended in an appropriate volume of liquid ice-cold Matrigel for passaging at a 1:3 ratio. The organoid/Matrigel mixture was dispensed (50 μL/well) in a pre-warmed 24-well plate, incubated for 5 min at 37 ˚C to solidify Matrigel domes and, subsequently, growth medium (500 μL/well) was added to cover the Matrigel domes. Typically, organoids were passaged every 3–4 days.

### Polarity reversion of organoids

#### Preparation of low-attachment plasticware

Low-attachment plasticware is essential for polarity-reverted ‘apical-out’ (AO) organoids: (1) to prevent adherence of the BO organoids to the plastic culture plates and loss of 3D morphology; (2) to prevent the non-specific adhesion of organoids to the plastic surfaces during handling. To prepare low-attachment centrifuge vials and 24-well culture plates, 0.5% lipidure-coating solution (95% molecular grade ethanol, made from the powder Amsbio, AMS.52000034GB1G) was added to cover the surface, followed by 1-5 min incubation at RT, under sterile conditions. Subsequently the coating solution was aspirated and the coated plastic surfaces were dried under a sterile air, and stored at RT until use.

#### ‘Apical-out’ (AO) organoid suspension culture in low-attachment plates

To produce AO organoids, we adapted the protocol of Co et al.^38^. Briefly, 1-3 day old cultures of BO organoids in 50 μL Matrigel domes at density of ~150–300 organoids/dome were rinsed with ice-cold 5 mM EDTA-PBS solution, followed by the dislodging of the Matrigel domes and resuspension by pipetting with 1 mL pipette tips (low binding tips; WB 5174S, Westburg) in a ice-cold 5 mM EDTA-PBS solution (500 μL/dome). Subsequently, the Matrigel/organoids suspension (pooled from 4 domes) was transferred into lipidure-coated 15 mL centrifuge vials and the volume was adjusted with cold EDTA-PBS solution to 12 mL. The suspension was incubated for 1 h at 4 °C with continuous gentle mixing to completely dissolve the Matrigel. Organoids were collected by centrifugation (300 g, 3 min, 4 °C), washed once with 10 mL ice-cold washing medium (DMEM high glucose GlutaMax^TM^ Supplement), followed by resuspension of the pellet in 3.2 mL of 37 °C pre-warmed HGM and dispensed in lipidure-coated (low-attachment) 24-well plate (400 μL per well). Organoids were cultured for 18-20 h to induce polarity reversion. AO organoid suspension culture could be cultured for up to 5 days.

#### Loading of AO organoids with dibutoxy-aza-BODIPY-doped nanoparticles and staining with fluorescent dyes

To evaluate the uptake of MNPs (Table 1), nanoparticles A-D were added to 1 day old AO organoid suspension culture in a low-attachment 24-well plate in HGM (AO organoid density at ~ 100-150 organoids/well, 400 μL medium, 10 μg/mL MNP) and incubated for 18-24 h prior to microscopy analysis.

For analysis of mitochondrial mobility, polarization, chemokine secretion and ATP, NP D was used at 10 μg/mL. To improve the yield of NP-loaded organoids, this was performed simultaneously with the polarity reversion. For the phasor-based counting method validation, NP D were added to organoids during the reversion procedure at 0, 0.01, 0.1, 1, 10, 25, 50 and 100 μg/mL in medium. For co-loading experiment, NP B and D were used individually (for a fingerprint analysis) and together at 10 μg/mL with the loading procedure done during the polarity reversion.

For the time lapse loading experiments, NP D (10 μg/mL) were added directly during monitoring to AO organoid suspension culture in “imaging medium” (“IM”, DMEM D5030 Sigma-Aldrich, without phenol red and sodium bicarbonate, supplemented with 10 mM D-glucose, 2 mM GlutaMax, 1 mM sodium pyruvate, 10 mM HEPES-Na, pH 7.2). Microscopy experiments were performed at different time points (0.5-24 h).

Where indicated, a 1 h co-staining with different fluorescent probes (see Table 2) was performed in HGM. Prior to the microscopy, organoids were washed 3 times to remove nanoparticles and other fluorescent probes by media exchange with the IM. For TMRM-FLIM^52^ experiments, 10 nM of TMRM was added to IM after the final media exchange and remained in solution during the imaging procedure.

#### Analysis of fixed organoids by immunofluorescence and phalloidin staining

Intestinal organoids (AO organoid suspension or BO organoids embedded in Matrigel) were washed 3 times with pre-warmed (37 °C) PBS, immediately fixed with warm 4% paraformaldehyde in PBS (10 min, RT), washed 5 times with PBS, permeabilized with 0.1% Triton-X100 in PBS (10 min, RT). Depending on the analysis, organoids were washed 5 times with PBS (for only phalloidin labeling) or TBST (20 mM Tris, 150 mM NaCl, 0.1% Tween-20, pH 7.6; for immunofluorescence combined with phalloidin labeling), incubated with a corresponding blocking solution (5% FBS / PBS or 5% FBS / TBST) and subsequently stained with Alexa Fluor 546 or Texas Red-conjugated phalloidin in blocking solution (1 h, RT). For immunofluorescence, fixed organoids were incubated 1 h with anti-ZO-1 mouse monoclonal primary antibody (Invitrogen, clone ZO1-1A12, 33-9100; final dilution 5 μg/mL), followed up by secondary anti-mouse IgG Alexa-Fluor Plus 488 conjugated antibodies (ThermoFisher Scientific, A32766) as described previously^24^ (see Table 2).

Stained organoids were washed 5 times with PBS and stored at 4 °C until analysis. The organoids were not embedded between glass slides to preserve their 3D organization.

For NP D concentration-dependent uptake study, organoids were fixed with 4% PFA as described above and stored at 4 °C in PBS until the analysis by microscopy.

#### Microscopy

Confocal FLIM microscopy was performed on a Stellaris 8 Falcon microscope, (Leica Microsystems, Ghent Light Microscopy Core, Ghent University), equipped with a white-light laser (440 − 790 nm), HC PL Apo 10×/0.4 air, HC Fluotar 25×/0.95 W, HC PL Apo 40×/1.25 GLYC corr, HC PL Apo 63×/1.4 oil objectives, HyD X, HyD R and HyD S detectors, temperature-/ CO_2_-controlled incubator, and dedicated LAS X acquisition and analysis software (ver. 4.6.0 and ver. 4.8.1), as described previously^26,69^. For routine microscopy, a 40×/1.25 GLYC corr. objective was used, with typical settings: 80 MHz white-light laser frequency pulse, scan speed 100-400 Hz, pixel dwell time 2.087-7.688 µs, pinhole 1.0-1.2 airy units, 1-3 frame repetition rate, 512 ×512 or 1024 ×1024 pixels resolution. The following general excitation and emission settings were used for fluorescence imaging (see Table 2): dibutoxy-aza-BODIPY-doped nanoparticles (MNP) excited at 698 nm (laser power 10%, emission 717 - 753 nm), TMRM excited at 540 nm (laser power 1%, emission 559 - 585 nm), WGA-Alexa Fluor 488 excited at 490 nm (laser power 4%, emission at 495 - 520 nm), UAE-Fluorescein conjugate excited at 495 nm (laser power 10.54%, emission at 497-568 nm), Nile Red excited at 490 nm (laser power 4%, emission collected at 588 - 620 nm), MTR excited at 581 nm (laser power 1.2%, emission collected at 594 - 654 nm), MTG excited at 490 nm (laser power 6.6%, emission at 497 -551 nm), phalloidin-Texas Red conjugate excited at 579 nm (laser power 3.21%, with emission collected at 588 - 649 nm).

Widefield fluorescence microscopy was performed on an inverted fluorescence microscope IX81 (Olympus-Evident), equipped with motorized Z-axis control, CoolLED pE4000 (16 channels, 365-770 nm), ORCA-Flash4.0LT+ (Hamamatsu) cMOS camera, temperature controlled stage (OkoLab), and air objectives 4x/0.13 UPlanFLN, 10x/0.3 UPlanFLN, 40x/0.6 LUCPlanFLN, water immersion objective 60x/1.0 LUMPLFLN, CellSens Dimension v.3 software as described previously^70,71^.

#### FLIM analysis of dibutoxy-aza-BODIPY-doped nanoparticles in solution

Dispersions of different nanoparticle types (A, B, C and D – 8 month in storage after production) were prepared by diluting stock nanoparticle dispersions to a final concentration of 500 μg/mL in MQ-grade water and 25% FBS and 50% imaging medium (for NP A and NP D). For phasor plot comparison in response to nanoparticle dilution, series of nanoparticle type D dilution in milli-Q grade water were performed over concentration range of 0.58-500 μg/mL. Milli-Q grade water was used as a negative control. Subsequently, confocal FLIM measurements of a 30 μL drop of nanoparticles dispersion on a cover glass surface were performed with HC PL Apo 10×/0.4 air, pinhole 1.0 AU and 1.16 ×1.16 mm field of view was imaged with 512 ×512 pixels resolution, 200 Hz scanning speed, 3 frame repetition per image. The frequency of the laser pulse was set to 80 MHz with the power of the laser excitation at 698 nm 8%. Pixel by pixel acquired fluorescence decays were automatically converted into phasor plot data in dedicated LAS X software (Leica Microsystems, version 4.6.0). The corresponding τ_ϕ_ (tau phase) of the phasor patterns was calculated from the center position of the circular region of interest (ROI) applied to the phasor plots. ROI-based color coding was applied to related pixels on the images. For comparing lifetimes of different nanoparticle types, the following phasor plot settings were used: first harmonic, threshold 7, median filter set as 3, bin 1, circular ROI radius 14. For the analysis of a dilution effect on nanoparticle type D lifetimes, the following phasor plot settings were used: first harmonic, threshold 4, median filter set as 11, nanoparticle signal circular ROI radius 58, with pixel binning 1 or 3.

For precise calculation of nanoparticle fluorescence lifetime, a 3-exponential reconvolution fitting model was applied to collected global decays and intensity weighted mean fluorescence lifetime (τ_m_) in LAS X 4.6.0 software (Leica Microsystems).

The fluorescence lifetime of nanoparticles (500 μg/mL in MQ-grade water; Figure S2d) was also measured after 20 months of storage, in comparison with non-encapsulated dibutoxy-aza-BODIPY (0.13 mg / mL, DMSO). Confocal FLIM were done with HC PL Apo 10×/0.4 air objective, pinhole 1.0 AU, field of view 0.397 ×0.397 mm, resolution 512×512, 400 Hz scanning speed, 3 frame repetition per image with different laser pulse frequency 80, 40 and 20 MHz with the power of the laser excitation at 698 nm 10%. Phasor plot analysis was done as described previously (using LAS X version 4.8.1) with : threshold 5, median filter 11, binning 1, first harmonic.

#### Comparison of intensity- and lifetime (phasor)-based analysis approaches

The experiment was repeated twice using the same organoid line. Organoids were loaded with NP D at various concentrations (0-100 µg/mL) during the polairty reversion and co-stained with WGA-Alexa Fluor 488 for determining topology and segmentation. Subsequently, they were fixed with 4% PFA, washed with PBS and stored at 4 °C in PBS, prior to microscopy. FLIM microscopy was performed using HC PL Apo 40×/1.25 GLYC corr objective, 1024 ×1024 pixel resolution, 1 frame repetition, 100 Hz scanning speed, pixel dwell time 7.7 μs, excitation at 490 nm (1.8-3.3% laser power, for WGA) and 698 nm (10% or 30% laser power intensity, as indicated, NP D), 80 MHz frequency laser pulse, zoom 1, one sequence excitation and emission light collection with HyD X (range 495-541 nm) and HyD R (717-753 nm) detectors on a confocal FLIM microscope. Each experimental group including control (unloaded organoids) contained 17-33 organoid FLIM images, acquired as described above. First, FLIM images of NP D fluorescence and corresponding control images were pre-analyzed in LAS X software (Leica Microsystems, version 4.6.0) to confirm that only phasor clusters in the 1.96 ns lifetime zone were present and no unrelated phasor clusters appeared on the control phasor plots images. Different median filter and pixel binning values were tested to decrease dispersion of the phasor clusters with reasonable preservation of the image spatial resolution. After choosing pixel binning 3 as the setting parameter for the phasor plots we optimized the intensity (not phasor) threshold in randomly screened images from the experimental groups at the lowest and highest loading concentrations. The threshold was selected to filter out the blurring pixels from the images of the high NP D loading concentration groups, while keeping the main pixels, reflecting the real NP signals inside cells, intact. Pre-analyzed raw organoid images were exported in PTU format and analyzed in napari viewer, with a FLIM phasor plotter plugin [GitHub - zoccoler/napari-flim-phasor-plotter], (10.5281/zenodo.12620955). Using WGA as a marker of organoid ROI selection the ROI mask was manually applied to the NP D fluorescence channel image and the ROI image was processed with application of pixel binning 3 and intensity threshold value 188 a.u., with the subsequent phasor plot reconstruction using median filter 10. Accordingly, all individual organoids on images were analyzed and their ROI area and total phasor plot points G and S coordinates were exported as csv table format files. To speed up the analysis a macros code ‘Supplementary Code area_GS_export’ (10.5281/zenodo.14742564) was produced in Python using Claude AI (www.claudeai.com) and validated with manually extracted and processed data. The content of all csv phasor plot coordinate files was pre-screened to remove nonsense point data (points where both G and S coordinates or one of them had value 0) and the total number of points from NP D lifetime zone was counted using Claude AI (‘Supplementary code_GScount’, 10.5281/zenodo.14742564). Subsequently, all the exported data (apical-basal organoid topology, organoid ROI square area, number of cluster points) were organized in Microsoft Excel and the number of points was normalized per organoid ROI square area to have a number of FLIM events per square unit. Accordingly, organoids were classified based on their apical-basal topology into AO and BO (include BO and AB organoids) organoids groups for all loading concentration groups.

In addition to the FLIM and data analysis, the same organoid suspension sample was imaged with widefield fluorescence microscope with air UPlanFLN, 10x/0.3 (experimental repeat R1) and UPlanFLN, 40x/0.6 (experimental repeat R2) LUCPlanFLN objectives (Olympus-Evident) as described above. The following acquisition settings were applied: 1024×1024 pixel resolution, NP D excited with 660 nm (LED power 40%) with emission collected at 705-845 nm (exposure time 40 ms) and WGA-Alexa Fluor 488 excited with 470 nm (LED power 40%) with emission collected at 510-550 nm (exposure time 7.363 ms). Images were exported as TIFF files and analyzed in Fiji software. The organoid ROI mask was made based on WGA-Alexa Fluor 488 segmentation and applied to NP D spectral channel fluorescence intensity image to extract total organoid intensity. The NP D background image intensity was calculated as an average from 10 random 1-pixel points surrounding organoids. Organoid ROI square areas, background and total organoid intensities were exported to Microsoft Excel table file and an individual organoid intensity values per area were calculated by substracting background intensity from the total organoid intensity value and normalizing by the ROI area.

Both FLIM events and intensity datasets were tested for normal distribution using normality test calculator [Georgiev G.Z., *“Normality Calculator”*, [online] Available at: https://www.gigacalculator.com/calculators/normality-test-calculator.php URL [Accessed Date: 24 Jan, 2025] using a sum of normality tests, including Shapiro-Wilk test / Shapiro-Francia test (*n* < 50 / *n* > 50), Anderson-Darling test, Jarque & Bera test, Cramer-von Mises test, d’Agostino-Pearson. The statistical analysis was done in OriginPro software with graphs made in Origin 6.0 (OriginLab). Accordingly, non-parametric tests were used: (i) Mann-Whitney for comparison between AO and BO organoids for each loading concentration data set (see Tables S3, S4, Fig. 4c, d) and (ii) Kruskal-Wallis ANOVA for multiple group comparison with Dunn’s and Conover’s post-hoc test analysis between individual concentration groups among AO and BO grouped organoids (see Tables S5-S8).

#### Phasor-FLIM-event counting approach for analysis of MNP mixtures

FLIM imaging data of control (no NP) and organoids loaded with NP D and B nanoparticles (as a mixture or separately) were exported as PTU files, processed with the napari-phasor-plotter plugin to extract G and S phasor point coordinates. After removing of nonsense points as described above, phasor coordinates were organized in Microsoft Excel table. The average values of G and S coordinates for individual NP D, NP B and MNP mixture-loaded organoids were counted and compared using Kruskal-Wallis ANOVA with Dunn’s post-hoc test. The fingerprint phasor clusters of NP D and NP B in organoids were reconstructed in Microsoft Excel from the sum of all corresponding phasor plots coordinates (see Fig. 5c). For every fingerprint cluster the values of average G coordinates (G_fave_) ± standard deviation (SD) were calculated and applied as thresholds for the pure NP D and B fluorescence lifetime zones. Similarly, a few phasor clusters of individual organoids (see Table S9, Fig. 5d) loaded with a MNP mixture were reconstructed and the percentage of pure D, pure B and mixed D/B intracellular MNP were counted by the attributing corresponding G coordinates to the following zones: G ≤ G_fDave_ + SD_D_ (NP D lifetime zone), G ≥ G_fBave_ – SD_B_ (NP B lifetime zone) and G_fBave_ – SD_B_ > G < G_fDave_ + SD_D_ (D / B mixed lifetime zone). The corresponding Claude AI generated code was used (‘Supplementary code_tau_zones’, 10.5281/zenodo.14742564).

#### Data assessment and Statistics

Origin software (ver 6.0 and 12) was used for statistics, except for mitochondrial dynamics and morphology. The detailed statistical analysis described in the corresponding sections of methods, figures and supplementary tables. Error bars and *p* values were reported in the figures and figure legends. We collected and analyzed all data objectively using instruments without bias and experimental samples were randomly chosen into groups.

## Supplementary information

Supplementary file in PDF

Video S1

Video S2

Video S3

Video S4

Video S5

Video S6

## Data Availability

Raw PTU and CSV files for analysis of organoids loaded with MNP mixtures by Phasor-FLIM-event counting approach are available at Zenodo (10.5281/zenodo.14742564). Custom codes used for GS export and GS coordinates analysis are available at Zenodo. Raw PTU files and processed CSV files for mitochondrial polarization phasor analysis in organoids are available at Zenodo (https://zenodo.org/records/14417182). Mitochondrial microscopy data, Nellie-processed csv files, and associated analysis plots generated using custom code are available at Zenodo (https://zenodo.org/records/14417688) and a public GitHub repository (https://github.com/HangZhouFLIM/FLIM_nano). Other data are available upon request.
