# Supplementary material for: Visualizing the internalization and biological impact of nanoplastics in live intestinal organoids by Fluorescence Lifetime Imaging Microscopy (FLIM)
Source: Light Sci Appl. 2025 Aug 12;14:272. doi: 10.1038/s41377-025-01949-0 (PMC12343940; doi:10.1038/s41377-025-01949-0)
Supplement: Supplementary file 1 — Supplementary file in PDF [file 41377_2025_1949_MOESM1_ESM.pdf]

Supplementary Information for:

Visualizing the internalization and biological impact of nanoplastics in live intestinal organoids by Fluorescence Lifetime Imaging Microscopy (FLIM)

Irina A. Okkelman[1,2$], Hang Zhou[1$], Sergey M. Borisov[3], Angela C. Debruyne[1], Austin E. Y. T. Lefebvre[4], Marcelo Leomil Zoccoler[5], Linglong Chen[6], Bert Devriendt[6*], Ruslan I. Dmitriev[1,2*]

[1]Tissue Engineering and Biomaterials Group, Department of Human Structure and Repair, Faculty of Medicine and Health Sciences, Ghent University, The Core, C. Heymanslaan 10, 9000, Ghent, Belgium.
[2]Ghent Light Microscopy Core, Ghent University, 9000 Ghent, Belgium
[3]Institute of Analytical Chemistry and Food Chemistry, Graz University of Technology, Stremayrgasse 9, Graz, 8010 Austria
[4]Calico Life Sciences LLC, South San Francisco, CA, 94080 USA
[5]Bio-Image Analysis Technology Development Group, DFG Cluster of Excellence “Physics of Life”, TU Dresden, 01307 Dresden, Germany
[6]Laboratory of Immunology, Department of Translational Physiology, Infectiology and Public Health, Faculty of Veterinary Medicine, Ghent University, 9820 Merelbeke, Belgium

[$]these authors contributed equally to this work

[*]To whom the correspondence should be addressed:
E-mail: Ruslan.dmitriev@ugent.be ; b.devriendt@ugent.be; Tel: +32-93325133

Supplementary figures S1-S15

Supplementary tables S1-S10

Supplementary videos (description) S1-S6

Supplementary methods

Supplementary figures S1-S15

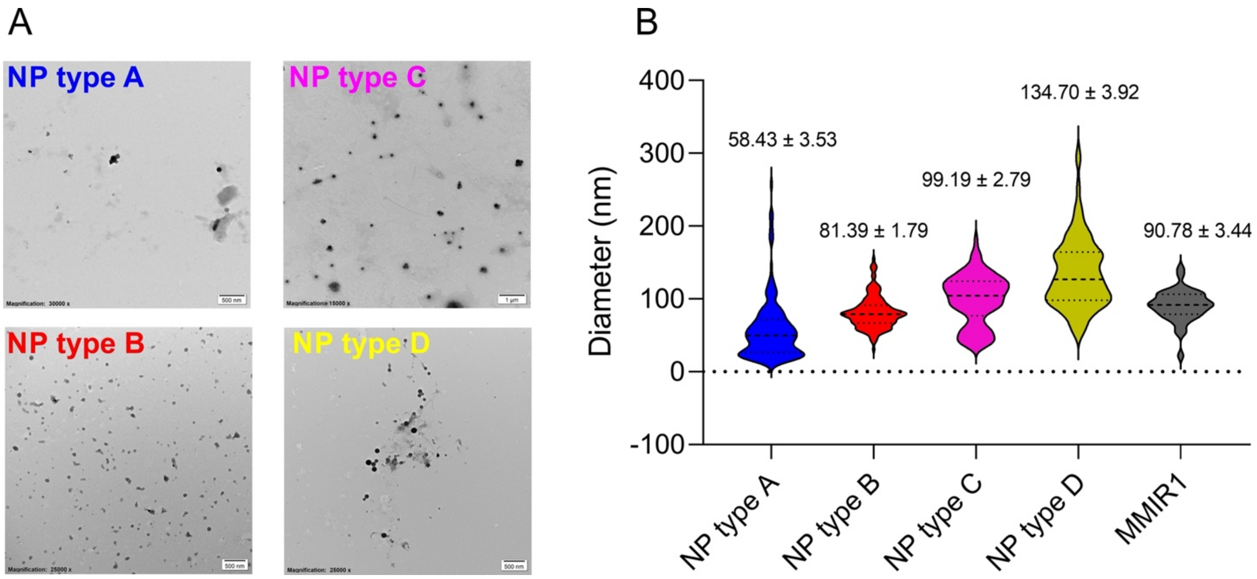

Figure S1. TEM and size distribution of the NP A-D. A: Transmission electron microscopy (TEM) of nanoplastic particles. Scale bar is 500 nm for type A, B and D and 1 µm for NP type C (1 µm). **B**: Size distribution of the nanoplastics type A-D with RL-100 based nanoparticles MMIR1 as control. Data shows the average size ± SEM for 150 counted nanoparticles and 50 for nanoparticle control.

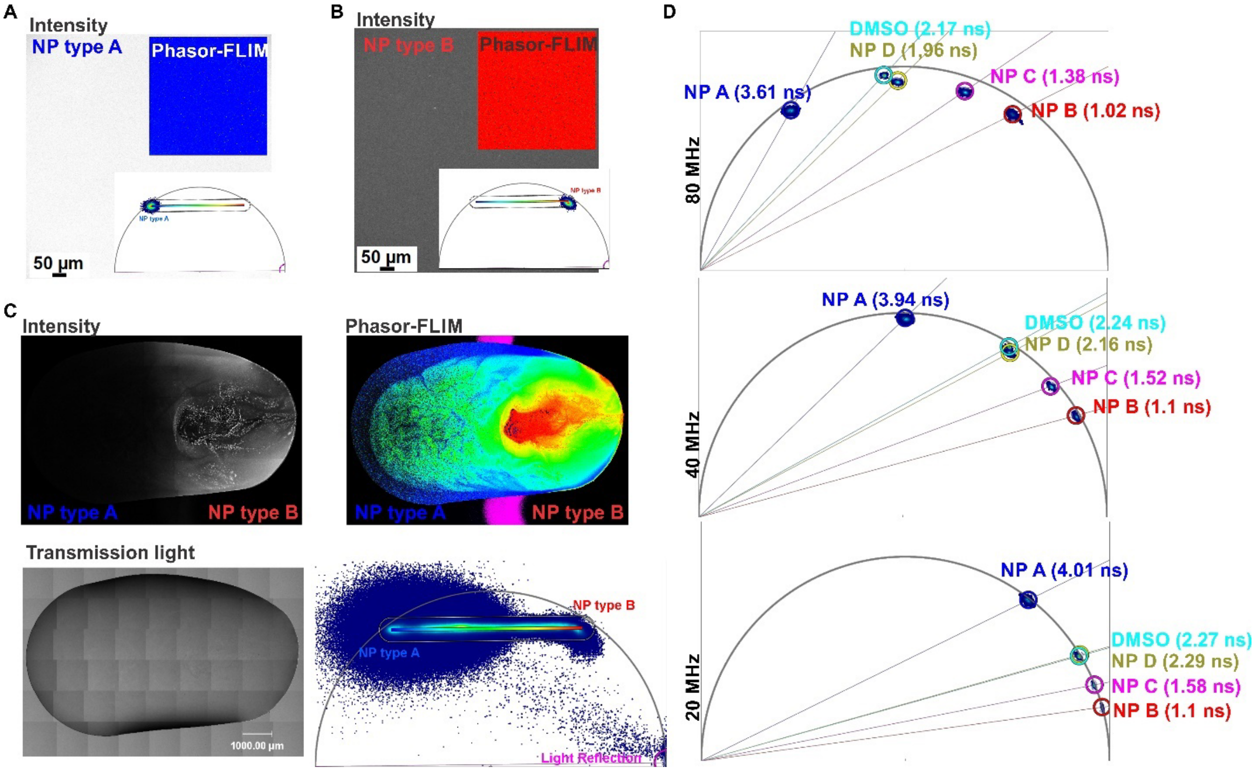

Figure S2. FLIM resolves different types of NP in aqueous dispersions. NP A and B were measured individually and in half-mixed water drops at 0.5 mg/ mL with the following analysis of the phasor patterns

changes. **A, B:** FLIM of pure NP A and NP B species prior to mixing. Raw intensity images (at the same scale range) are shown together with the color mask applied intensity images. False color coding applied as shown on the corresponding phasor plots with a range of τ_ϕ from 3.7 (blue) to 1 (red) ns. **C:** Large field of view mosaic FLIM of the half-mixed solutions of NP A and B. The raw intensity and phasor-mask applied intensity images are shown together with the corresponding transmission light image of the combined (half-mixed drops of NP A and B water solutions). Resulting mosaic phasor pattern (with the corresponding ROI mask – on the top and the raw phasor – on the bottom) localized on a line between opposite phasor positions of the individual unmixed NP A and B species. **D**: Phasor-FLIM characterization of NP A-D (20 months of storage after production, 0.5 mg / mL, in deionized water, 37 ^{o}C; blue, yellow, magenta and red circular phasor ROI masks) and the free dibutoxy-aza-BODIPY dye in DMSO (0.13 mg / mL, 37 ^{o}C; cyan circular phasor ROI mask). Corresponding fluorescence decays were collected with 80, 40 and 20 MHz laser pulse frequency. Centre τ_ϕ values are indicated in brackets.

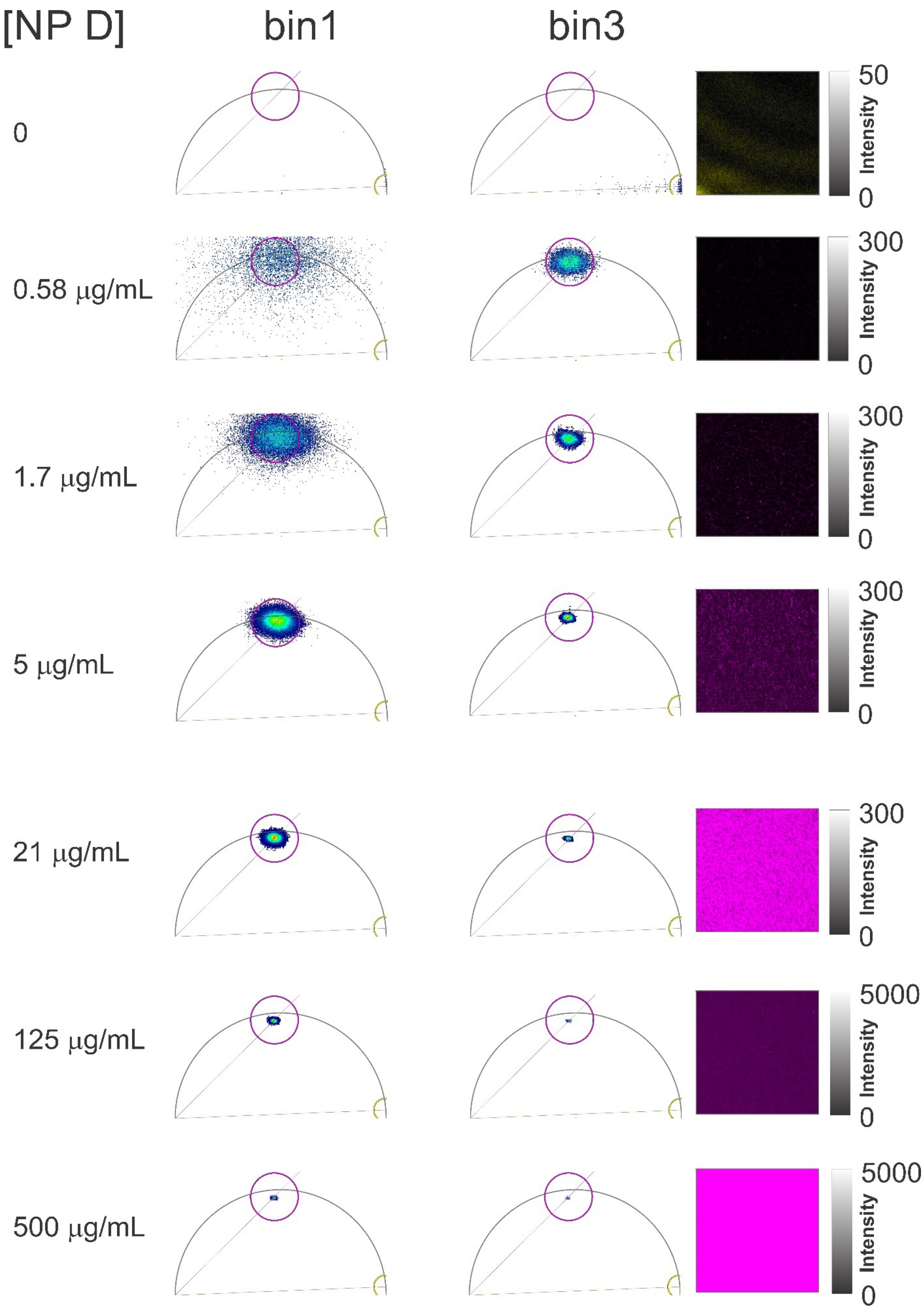
[NP D]
bin1
bin3
0
0.58 μg/mL
1.7 μg/mL
5 μg/mL
21 μg/mL
125 μg/mL
500 μg/mL
50
300
5000
Intensity
0

Figure S3. Pixel binning decreases scattering, noise and NP D signal phasor patterns on a phasor plot. The imaging was done with 200 Hz scanning speed, 3 frame repetition, 512 x 512 resolution, pinhole 1 AU. Phasor analysis of series of NP D dilutions in water was performed with LAS X version 4.6.0 (Leica Microsystems) software: harmonic 1, median filter 11, threshold 4, pixel binning 1 or 3. Magenta circular ROI marks the estimated localization of NP D related pixels ($\tau_{\phi} = 2.023$ ns, radius 58), yellow circular ROI marks the estimated localization of noise pixels ($\tau_{\phi} = 0.083$ ns, radius 28). Examples of fluorescence intensity images (for pixel binning 3) of noise (the water drop) and NP D dilution presented on the right with the applied false color masks based on corresponding phasor plot ROIs (on the left).

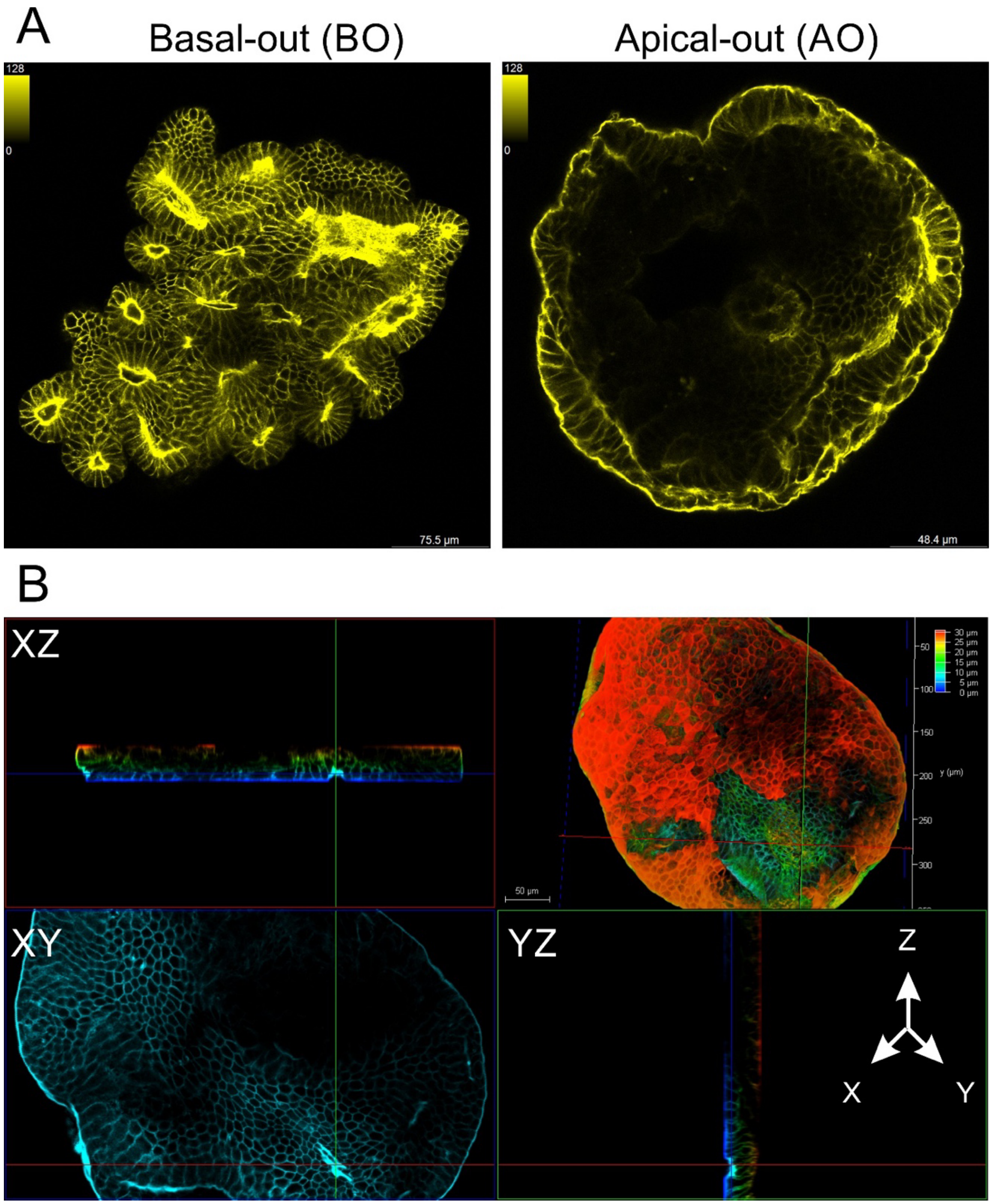

Figure S4. Representative confocal fluorescence microscopy images of polarity reverted (AO) and BO organoids, with F-actin labeling of apical membrane with phalloidin-Alexa 546 conjugate. A: XY optical sections of BO (Matrigel embedded organoid) and AO (1-day old suspension of organoids on a low attachment surface) pig intestinal organoids. **B:** Depth color-coded 3D reconstruction of AO organoid, imaged in glass mounted sample. Scale bars are indicated.

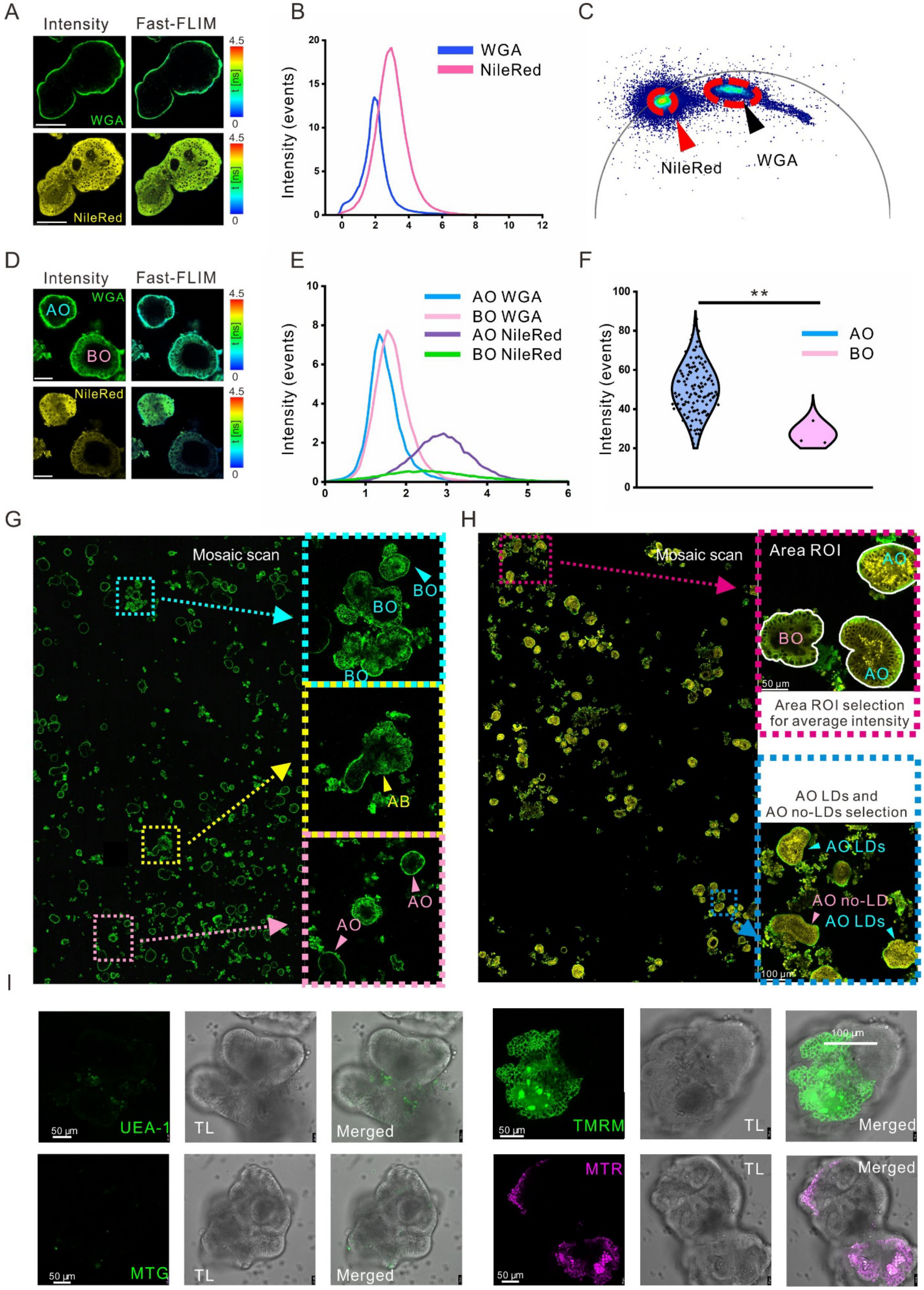
A
Intensity
Fast-FLIM
WGA
NileRed
B
WGA
NileRed
Intensity (events)
C
NileRed
WGA
D
Intensity
Fast-FLIM
AO
BO
WGA
NileRed
E
AO WGA
BO WGA
AO NileRed
BO NileRed
Intensity (events)
F
**
AO
BO
Intensity (events)
G
Mosaic scan
BO
AB
AO
H
Mosaic scan
Area ROI
50 µm
Area ROI selection for average intensity
AO LDs and AO no-LDs selection
AO LDs
AO no-LD
100 µm
I
UEA-1
TL
Merged
MTG
TMRM
MTR
50 µm
100 µm

Figure S5. Evaluation of live organoid staining with WGA-Alexa Fluor 488, Nile Red and other live imaging dyes. A: Representative confocal fluorescence intensity and fast-FLIM images of an apical-out porcine small intestinal organoid labeled with WGA and Nile Red. Scale bar is 100 μm. **B, C:** Fluorescence lifetime distribution histograms and phasor plot for WGA and Nile Red from the apical-out organoid shown in A (WGA ~2.1 ns, Nile Red ~3.3 ns). **D, E:** Comparison of WGA and Nile Red fluorescence intensity (D) and normalized lifetime distribution (E) in AO and BO organoids. Scale bar is 50 μm. **F:** Violin plots display average Nile Red fluorescence intensity in AO and BO organoids (AO area ROI counts: 119, BO area ROI counts: 3, intensity measured in FIJI software, p=0.006). **G:** WGA staining identifies three types of small intestinal organoids. Left: large mosaic scan of organoids in a single well, *Right:* representative images of BO, AB and AO from the mosaic scan. **H:** Analysis of WGA and Nile Red co-stained organoids. *Left:* Mosaic scan image. *Top right*: Organoid area ROI selection for average Nile Red intensity measurement using freehand tool in FIJI software. *Bottom right:* Counting the number of AO with lipid droplets (AO LDs) and without lipid droplets (AO no-LDs). **I:** Representative images of staining of polarity-reverted small intestinal organoids with UEA-1, MitoTracker Green (MTG), TMRM and MitoTracker Red (MTR) (indicated with green or magenta colors). TL: transmission light.

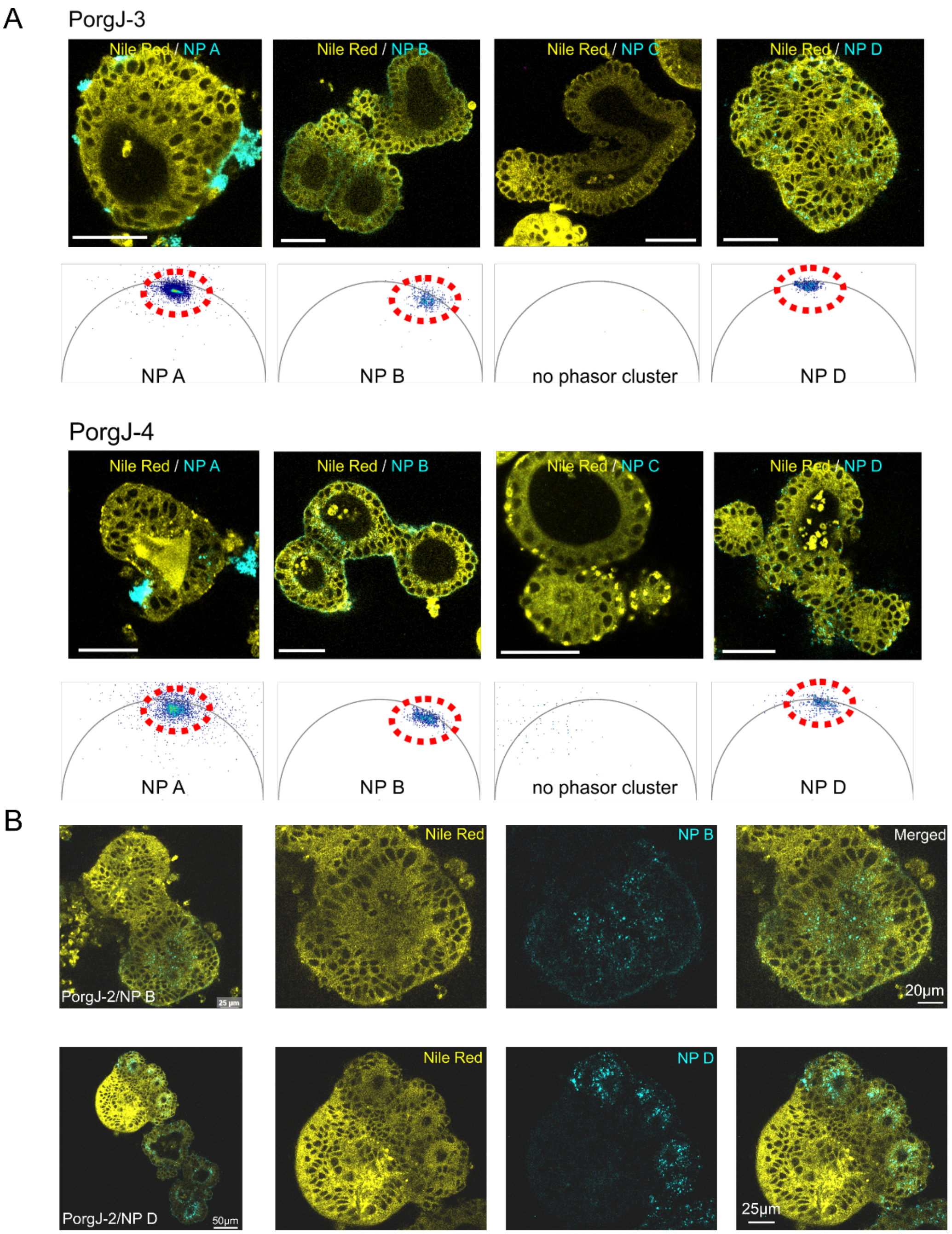

Figure S6. **Reproducibility of MNP uptake patterns across independent porcine intestinal organoid lines and high magnification images of NP B and D in live organoids. A**: Two additional independent porcine intestinal organoid lines (PorgJ-3 and PorgJ-4) were exposed to 4 NP types (A-D, shown in blue) and co-stained with Nile Red (shown in yellow). Both lines displayed similar uptake patterns to PorgJ-2: NP A displayed apical membrane accumulation, NP B and NP D displayed intracellular uptake, confirmed with phasor FLIM analysis. Scale bar is 50 µm. **B**: Magnified view of PorgJ-2 treated with NP B and NP D (shown in blue) and co-labelled with Nile Red (shown in yellow) confirms intra-organoid NP uptake.

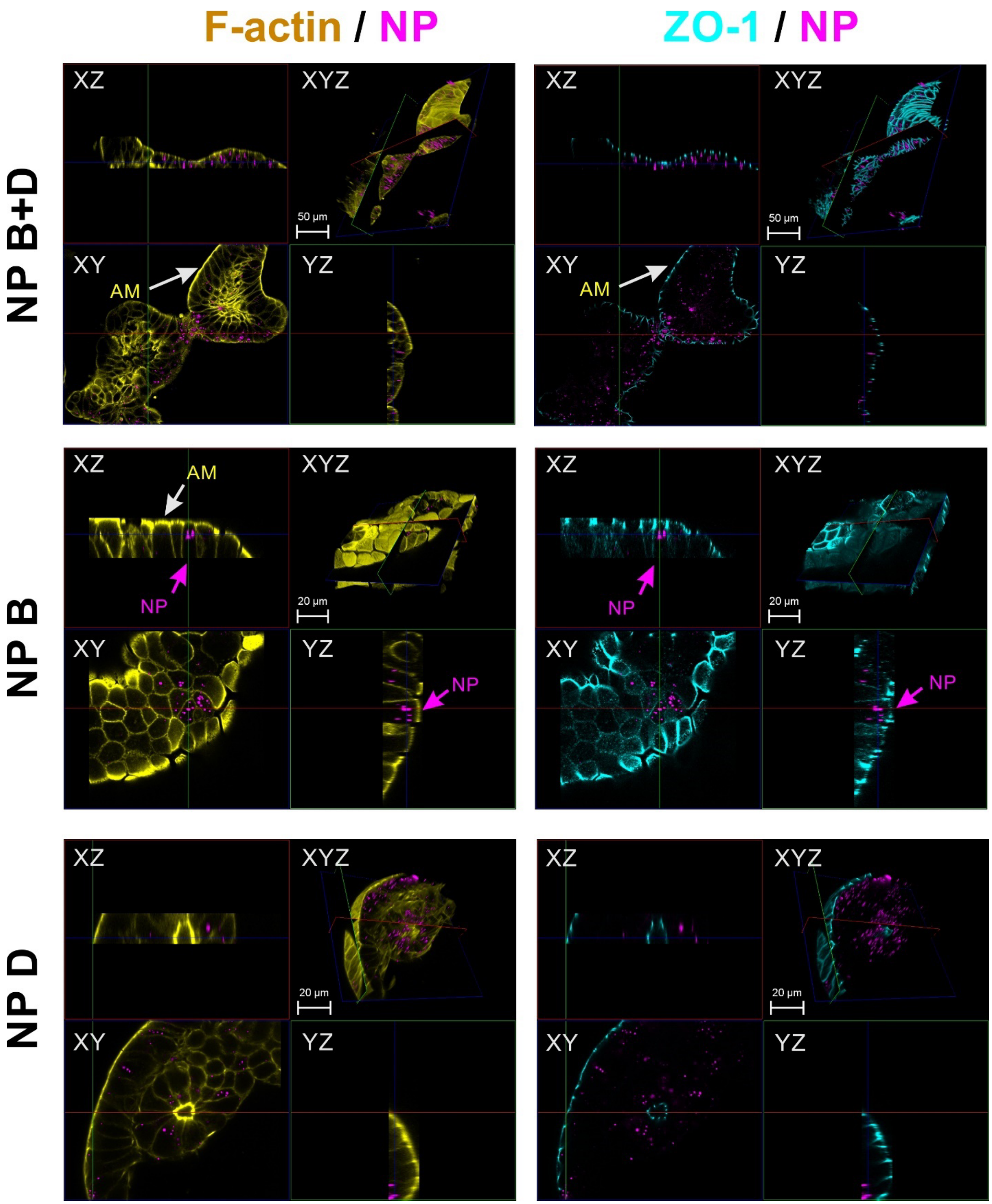

Figure S7. 3D confocal microscopy of F-actin organization (phalloidin labeling) and tight junctions (ZO-1) in AO organoids loaded with NP D and NP B, together or separately. Organoids were incubated with nanoparticles during polarity reversion (18 h), PFA fixed and immunostained with anti-ZO1 antibody / secondary Alexa-Fluor 488 antibody and phalloidin-Texas Red conjugate. Z stack (13 - 36 µm, step size 0.3 - 0.5 µm) images were produced with 400 Hz scan speed (pixel dwell time 1.4 µs), 1024 x 1024

resolution (pixel size: NP B+D 0.29 x 0.29 µm, NP D 0.09 x 0.09 µm, NP B 0.08 x 0.08 µm), 1 frame repetition). Cross-sections (slice views) and 3D reconstructions are indicated. White arrows point on the apical membrane (A))-out topology and magenta arrows point on NP localization inside individual cells. ZO-1 staining shows a characteristic AO shifted topology.

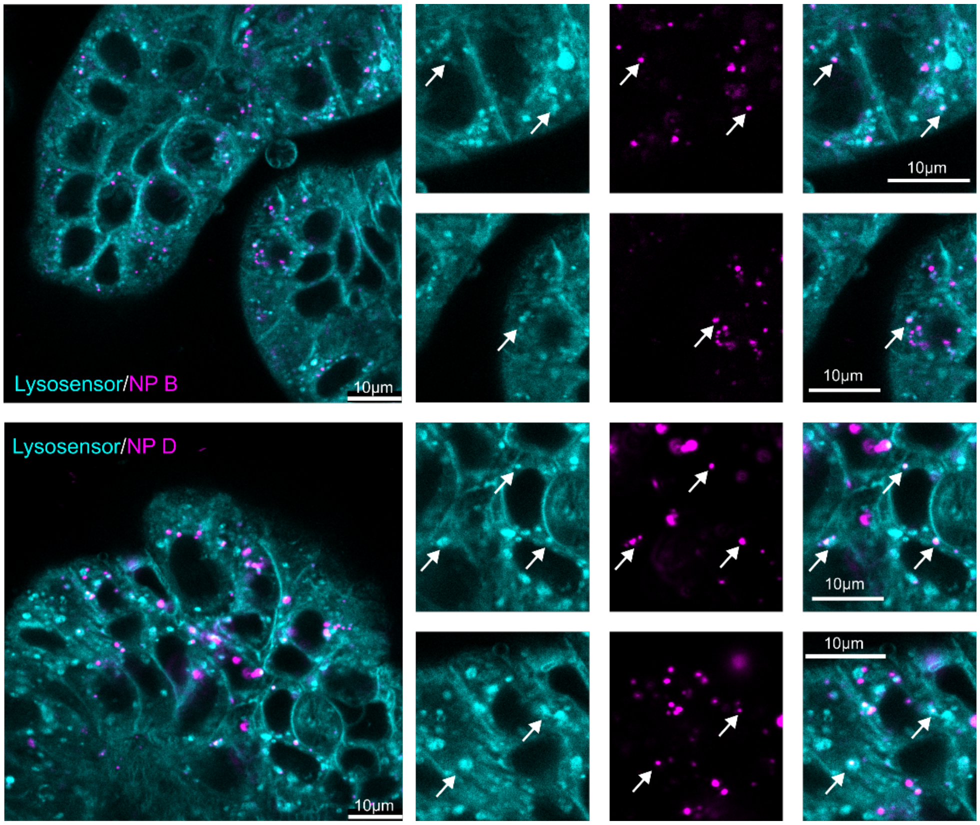

Figure S8. Lysosomal colocalization with nanoparticles B and D in live apical-out intestinal organoids. Representative confocal fluorescence intensity images of LysoSensor Green (cyan), nanoparticles (magenta), and merged channels. White arrows indicate NP colocalization with lysosomes; scale bar: 10 µm. Organoids were incubated with nanoparticles during polarity reversion (18 h), subsequently stained for 1 h with 2 µM LysoSensor Green DND-189 and imaged by confocal microscopy (exc. 448 nm 0.9%) with 1048 x 1048 resolution (76.34 x 76.34 µm physical size, pixel size 0.073 x 0.073 µm), 1 AU, 400 Hz scanning speed (pixel dwell time 1.363 µs), 1 frame repetition.

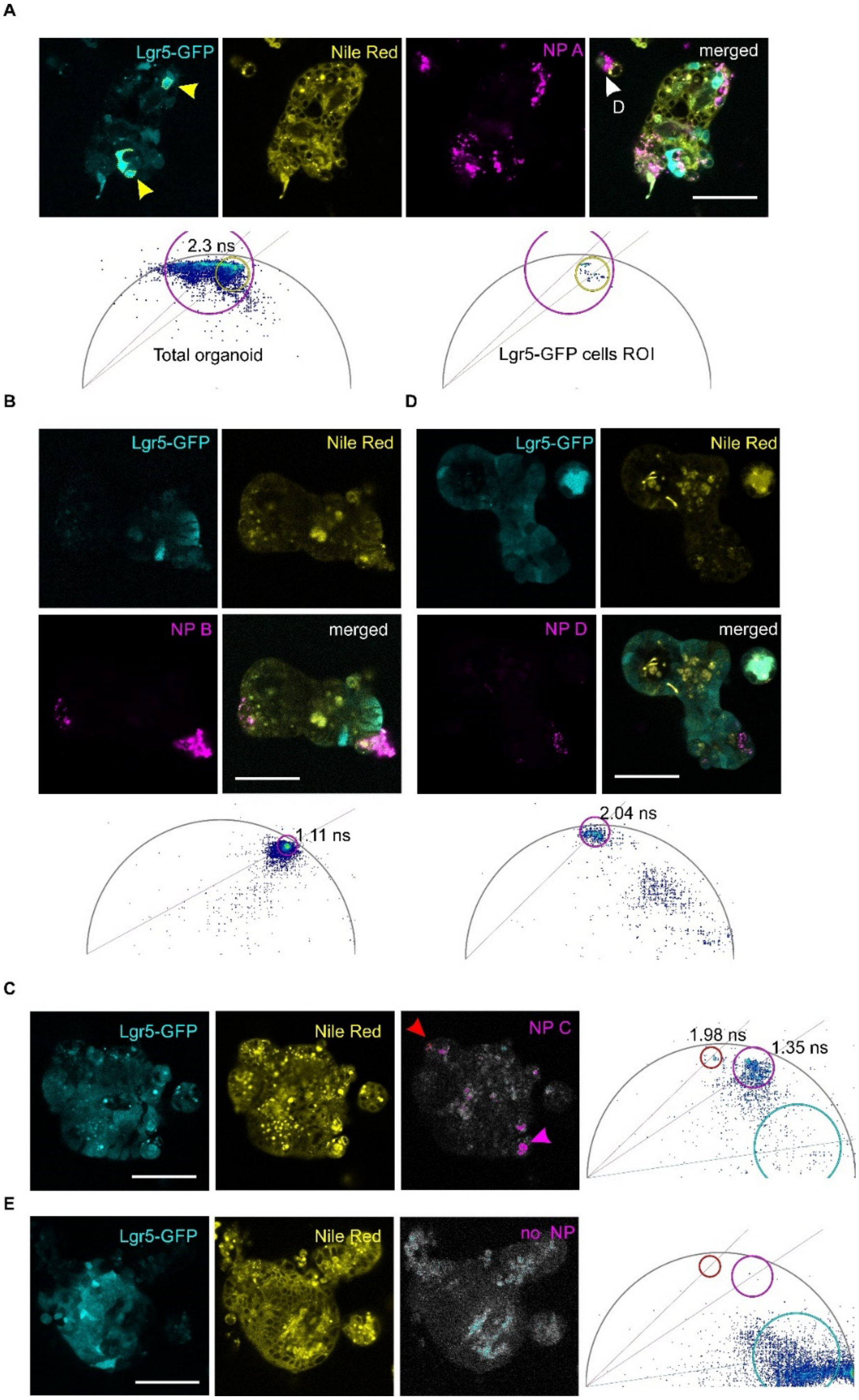

Figure S9. **Analysis of MNP uptake in polarity-reverted mouse Lgr5-GFP small intestinal organoids.** Organoids were polarity-reverted and incubated with NP A-D, essentially as with pig intestinal organoids (see Methods section). Lgr5-GFP organoids display expression of Lgr5-GFP (stem cell marker, shown in cyan), which disappears in differentiated cells (partially co-stained with Nile Red, yellow). NPs are shown in magenta. **A:** Local NP A uptake by specialized cell clusters, representative GFP negative or weak staining in organoids. NP A signal was also detected in cell debris, shown with white arrow (D). Yellow arrows indicate highly intense Lgr5-GFP positive cells chosen for ROI-based phasor analysis (yellow

circular ROI on the corresponding phasor plot). Magenta circular ROI on the phasor plot shows overall NP A cluster. **B, C, D:** NP types B, C and D uptake was observed only in specialized cell clusters of differentiated cells. **C:** NP type C uptake was detected by appearance of unique phasor clusters (red and magenta circular ROI on the phasor plot correspond to arrows and red and magenta color mask on the NP C intensity image) with characteristic fluorescence lifetime (τ_{ϕ} range of 1.98-1.35 ns). **E:** Unstained control group organoids did not display fluorescence with the characteristic lifetime of NP C (no phasor clusters in the red and magenta circular ROIs on the phasor plot). Cyan circular phasor ROI corresponds to intrinsic fluorescence noise signal of organoids in the spectral channel of dibutoxy-aza-BODIPY dye. Phasor plot was made as a sum of 8 phasor plots from corresponding FLIM microscopy data of individual organoids from the control group. Phasor plots from NP A-D were reconstructed from single organoid FLIM data set (per image) in LAS X software. with threshold 7, pixel binning 1 and median filter 11. Scale bar is 50 µm.

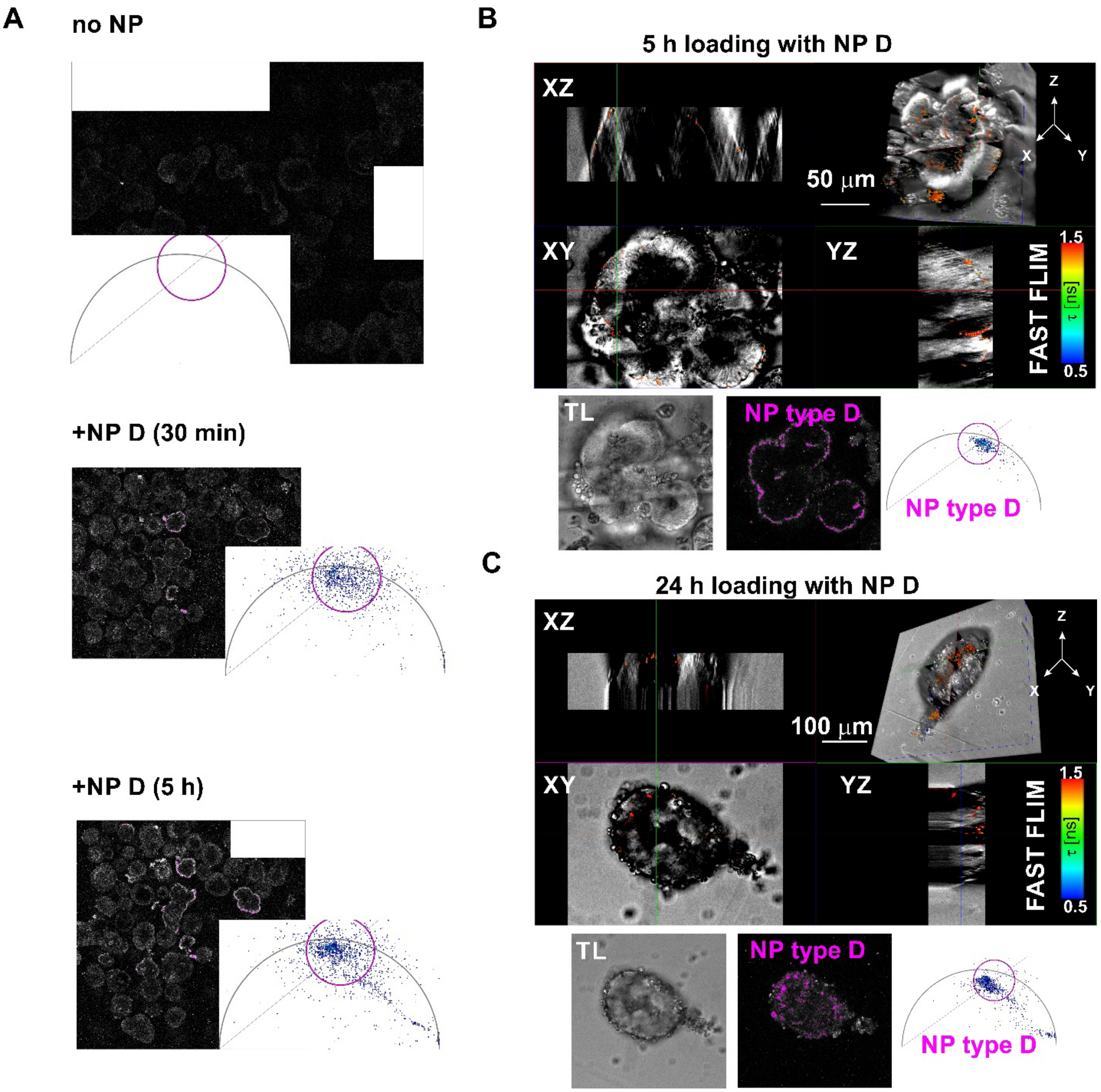

Figure S10. Time-course analysis of NP D uptake in pig intestinal organoids. Organoids were incubated with NP D and measured in fluorescence and transmission light channels with mosaic scanning (all the scans were performed within < 15 min timeframe). **A:** Mosaic images and corresponding phasor plots of control (no NP) and NP D-treated (0.5 - 5 h) organoid cultures. Circular magenta phasor ROI (with τ_ϕ ~ 2 ns) indicates the corresponding phasor cluster of NP D. **B, C:** 3D reconstruction and phasor plot of individual (middle) optical section of organoid analyzed at 5 h (B) and 24 h (C) time points. At 5 h point NP D fluorescence signal was mainly detected at the organoid periphery. At 24 h time point, NP D fluorescence signal was mainly detected inside of the organoid.

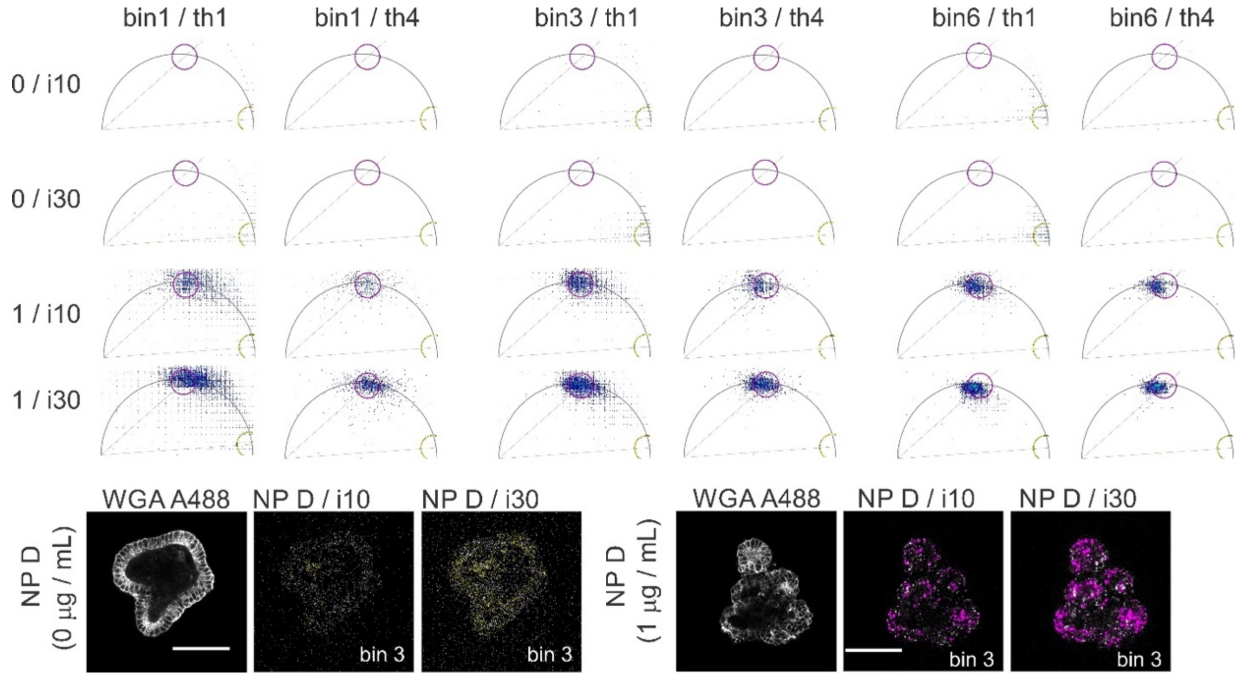

Figure S11. Effect of excitation laser power intensity, pixel binning and threshold application on the phasor clusters of NP D in pig intestinal organoids. Phasor plots were produced in LAS X software from unstained (referred as 0) or 1 µg / mL NP D-loaded (referred as 1) organoids images (the bottom row) acquired with either 10 % (i10) or 30 % (i30) laser power intensity. Pixel binning (bin) 1, 3 or 6 or phasor threshold (th) 1 or 4 were applied during image phasor plots reconstruction. Magenta and yellow circular phasor ROIs point at the position of the typical NP D cluster or the noise signal cluster, respectively. Yellow and magenta pseudocolor masks on the intensity images of NP D spectral channel correspond to lifetime events from the noise and NP D fluorescence signal phasor ROIs. Images were acquired with 100 Hz scanning speed, 1024 x 1024 resolution, pinhole 1 AU, 80 MHz laser pulse frequency, 1 frame repetition. rate. Median filter 11, harmonic 1 were applied for phasor plot reconstruction. Scale bar is 50 µm.

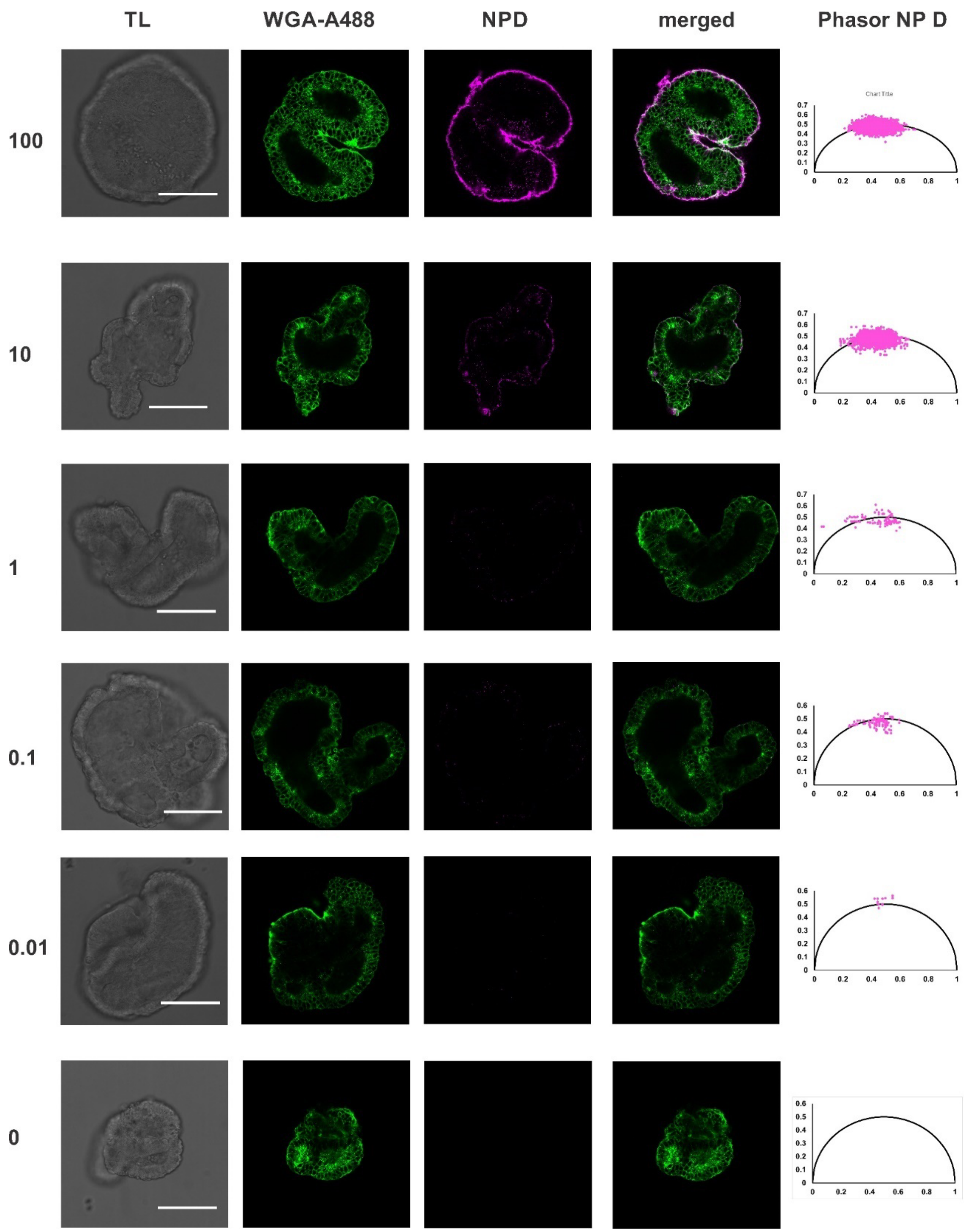

Figure S12. Typical examples of BO pig intestinal organoids loaded with NP D at various concentrations (0, 0.01, 0.1, 1, 10 and 100 µg/mL) and co-stained with WGA-Alexa Fluor 488. Images were acquired with pixel resolution 1024 x 1024, frame repetition 1, 100 Hz, pinhole 1 AU, 80 MHz pulse repetition rate. Scale bar is 50 µm. Corresponding phasor plots were exported from phasor FLIM napari plugin as a list of G and S coordinates and reconstructed in Microsoft Excel software. Phasor plots demonstrate that the number of acquired events in NP D lifetime zone clusters decreases proportionally with the decrease in NP D loading concentration down to zero event count for unstained organoid.

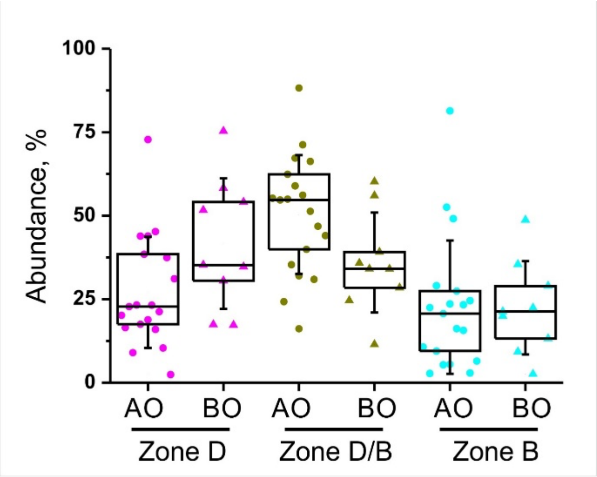

Fig. S13. Lifetime zone analysis of phasor clusters of AO and BO (including ABO) organoids loaded with a mixture of D and B NP. Boxes show 25 and 75 percentiles, while whiskers show standard deviation. Each point corresponds to individual organoid value. Mann-Whitney test was performed between AO (N = 19) and BO (N = 9) organoids at significance level $p < 0.05$. No statistical difference in abundancy of different lifetime zones of phasor clusters was found (see Table S9 for details).

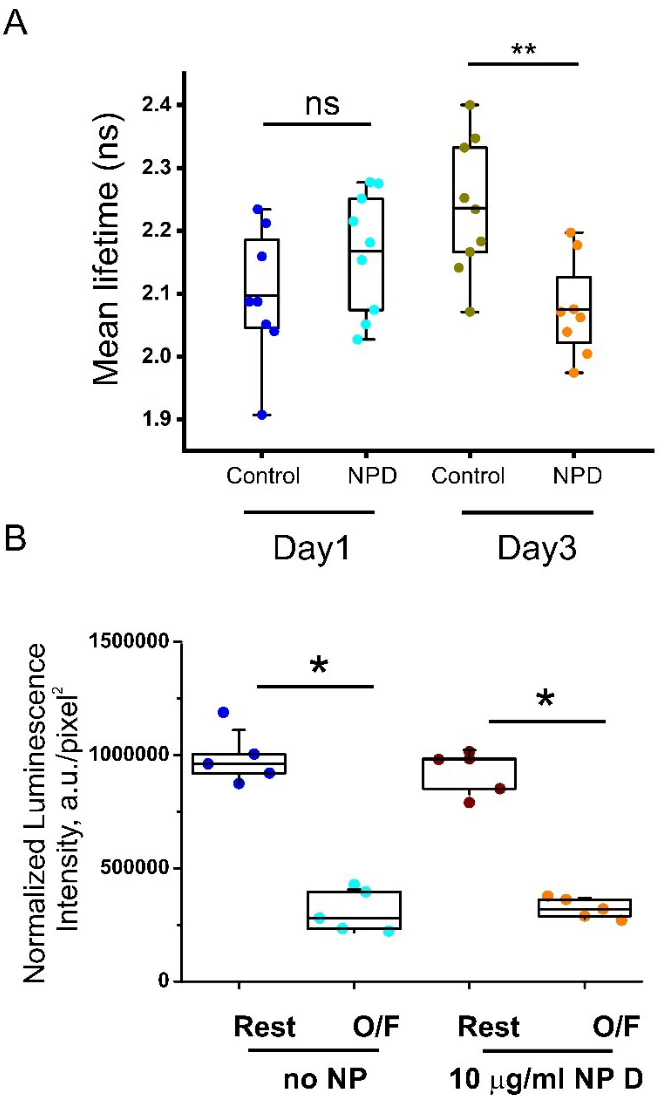

Figure S14. Independent experimental repeats of mitochondrial polarization and total ATP analysis in control (no NP) and NP D groups. A: TMRM fluorescence lifetime-based analysis of mitochondria polarization in BO organoids. Each point on a box plot represents the average TMRM τ_m value of each individual organoid. Box plots correspond to 25 and 75 percentiles; whiskers correspond to 10 and 90 percentiles. No significant difference was observed after 1-day exposure to NP D (N_{cont} = 8, N_{NPD} = 9 ; Mann-Whitney test, significance level $p < 0.05$). Mitochondrial polarization was significantly different (N_{cont} = 9, N_{NPD} = 8; Mann-Whitney test, ** $p < 0.01$) after 3 days exposure to NP D. Difference in TMRM fluorescence lifetime between control and NP D-treated group was 0.16 ns. **B**: Total cellular ATP analysis of control and NP D-treated organoids. No significant difference was observed between groups at resting (Rest) and under stimulation with Oligomycin / FCCP (O/F). In comparison, ATP levels were significantly different between Rest and O/F conditions inside each individual organoid group, reflecting organoid response to F/O inhibition of ATP production. Box plots correspond to 25 and 75 percentiles; whiskers represent standard deviation, asterisks point on groups with significant difference (N = 5, Mann-Whitney test, significance level $p < 0.05$).

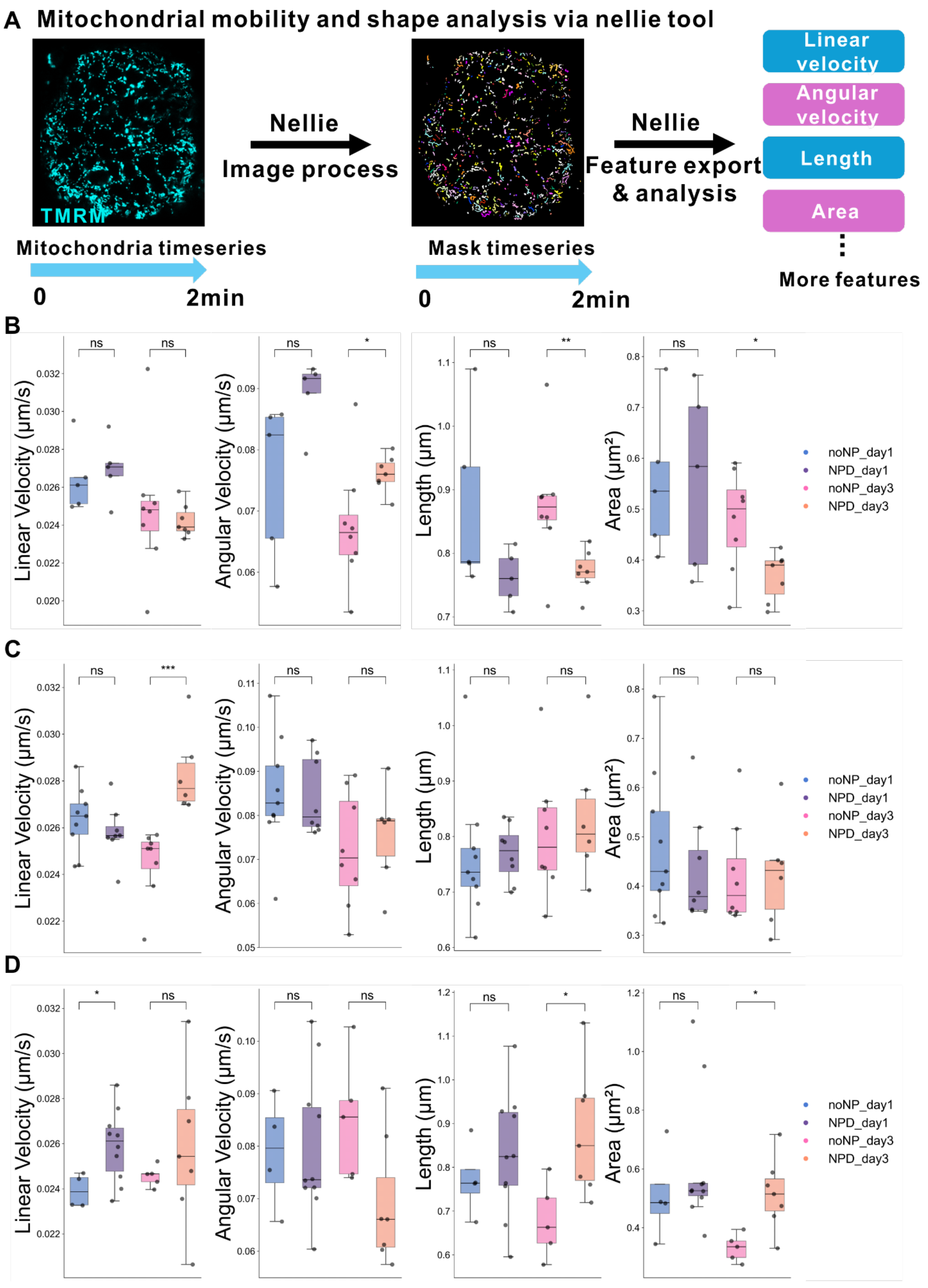
A Mitochondrial mobility and shape analysis via nellie tool
TMRM
Nellie
Image process
Nellie
Feature export & analysis
Linear velocity
Angular velocity
Length
Area
More features
Mitochondria timeseries
0
2min
Mask timeseries
0
2min
B
C
D
Linear Velocity (μm/s)
Angular Velocity (μm/s)
Length (μm)
Area (μm²)
ns
*
**
***
noNP_day1
NPD_day1
noNP_day3
NPD_day3

Figure S15. Effects of NP D on mitochondrial morphology and dynamics in pig intestinal organoids. **A:** The scheme illustrates the mitochondrial analysis workflow with Nellie software, used to process *XYt* time-lapse images (2 minutes, 47 timeframes) of TMRM fluorescence. **B, C, D:** Quantitative analysis of mitochondria features in organoids after 1- and 3-days exposure to NP D made for three independent experimental repeats. Box plots display average values of mitochondrial metrics per organoid (individual points represent single organoid unit, B: N_{noNP_day1} = 5, N_{NPD_day1} = 5, N_{noNP_day3} = 8, N_{NPD_day3} = 7, C: N_{noNP_day1} = 9, N_{NPD_day1} = 8, N_{noNP_day3} = 8, N_{NPD_day3} = 6, D: N_{noNP_day1} = 4, N_{NPD_day1} = 10, N_{noNP_day3} = 5, N_{NPD_day3} = 7, Mann-Whitney test, significance level ns p > 0.05, *p < 0.05, ** p < 0.01, ***p < 0.001).

Supplementary tables S1-S10

Table S1. Analysis of organoid polarity phenotypes in population of organoids in suspension from 5 independent experiments on polarity reversion. Total analysis of organoid population was done by mosaic scan imaging based on WGA-Alexa Fluor 488 conjugate staining of live organoids.

	**Total amount of organoids analyzed**	**Percentage of AO organoids from total organoid number**	**Percentage of AB organoids from total organoid number**	**Percentage of BO organoids from total organoid number**
1	136	96.3%	0.7%	3%
2	156	94.2%	1.9%	3.8%
3	102	98%	0%	2%
4	173	98.8%	0%	1.2%
5	98	98%	1%	1%

Table S2. The number and percentage of AO, partial AO and BO observed in 4 mosaic scan images, calculated based on WGA and F-actin staining patterns separately, as shown in Figure 2C.

Repeat	**Numbers of AO**	**Numbers of AB**	**Numbers of BO**	**Total**	**AO percentage**	**AB percentage**	**BO percentage**

1	WGA	60	5	0	65	92.3%	7.7%	0%
	F-actin	63	3	0	66	95.4%	4.5%	0%
2	WGA	79	7	1	87	90.9%	8%	1.1%
	F-actin	82	5	1	88	93.1%	5.7%	1.2%
3	WGA	98	6	1	105	93.3%	5.7%	1%
	F-actin	100	4	1	105	95.2%	3.8%	1%
. 4	WGA	116	5	0	121	95.9%	4.1%	0%
	F-actin	119	2	0	121	98.3%	1.7%	0%

Average topology type percentage ± SD (WGA- / F-actin labeling-based analysis): AO - 93.1± 2.11 % / 95.5 ± 2.13 %, BO - 0.53 ± 0.6 % / 0.55 ± 0.64 %, AB – 6.37 ± 1.82 % / 3.92 ±1.67 %)

Table S3. Comparison of NP D uptake in AO and BO organoids over a range of concentrations (0-100 μg/mL) using fluorescence intensity and Phasor FLIM events counting approaches. AO and BO organoid data from each concentration group were compared with Mann-Whitney test (at p = 0.05). Data represents one of two individual experimental replicates (R2), shown in Fig. 4 C,D.

**Intensity-based analysis**									
[NP D], μg / mL	N1 (AO) N2 (BO)	U	Z	Exact prob>|U|	Asymp. Prob >|U|	Median, (AO/BO)	Mean Rank (AO/BO)	Sum Rank (AO/BO)	Organoid loading events, % (AO/BO)
noNP	N1 = 15 N2 = 13	57	-1.8426	0.06475	0.06539	52.1 / 64.7	11.8 / 17.6	177 / 229	n/a
0.01	N1 = 19 N2 = 13	47	-2.9161	0.00259	0.00354	77.8 / 91.4	12.5 / 22.4	237 / 291	n/a
0.1	N1 = 16	21	-4.5679	<0.0001	<0.0001	91.5 / 177.9	9.8 / 26.5	157 / 584	n/a

	N2 = 22								
1	N1 = 17 N2 = 17	48	-3.3066	5.56E-04	9.44E-04	59.7 / 155.7	11.8 / 23.2	201 / 394	n/a
10	N1 = 25 N2 = 17	26	-4.7662	<0.0001	<0.0001	186.5 / 1791.7	14 / 32.5	351 / 552	n/a
25	N1 = 23 N2 = 19	59	-4.018	<0.0001	<0.0001	289.4 / 2462.8	14.6 / 29.9	335 / 568	n/a
50	N1 = 16 N2 = 20	20	-4.4411	<0.0001	<0.0001	501 / 5484.7	9.8 / 25.5	156 / 510	n/a
100	N1 = 17 N2 = 12	5	-4.2731	<0.0001	<0.0001	1653.3 / 12039.2	9.3 / 23.1	158 /277	n/a
**Phasor FLIM event counting analysis**									
[NPD], µg / mL	N1 (AO) N2 (BO)	U	Z	Exact prob>|U|	Asymp. Prob >|U|	Median, (AO/BO)	Mean Rank (AO/BO)	Sum Rank (AO/BO)	Organoid loading events, % (AO/BO)
noNP	N1 = 13 N2 = 9	58.5	0		1	0 /0	11.5 / 1..5	149.5 / 103.5	0 / 0
0.01	N1 = 21 N2 = 10	58	-2.8487	0.00508	0.0022	0 / 2.13644E-5	13.8 / 20.7	289 / 207	4.8 / 50
0.1	N1 = 13 N2 = 17	13	-4.0981	<0.0001	<0.0001	0 / 5.81784E-4	8 /21.2	104 / 361	38.5 / 100
1	N1 = 14 N2 = 9	2	-4.1128	<0.0001	<0.0001	0 / 1.2E-3	7.6 / 18.8	107 /169	14.3 /100
10	N1 = 10 N2 = 7	0	-3.3877	1.03E-04	7.05E-04	2.44E-3 / 2.508E-2	5.5 / 14	55 /98	60 / 100
25	N1 = 17 N2 = 12	26	-3.3436	3.95E-04	8.27E-04	2.07E-3 / 1.114E-2	10.5 /21.3	179 /256	88.2 /100
50	N1 = 10	0	-3.8378	<0.0001	<0.0001	2.06E-3 / 8.34E-2	5.5 / 16	55 / 176	100 / 100

	N2 = 11								
100	N1 = 15 N2 = 11	20	-3.2178	3.24E-04	6.46E-04	1.105E-2 / 0.16887	9.3 / 19.2	140 / 211	100 / 100

Table S4. Comparison of NP D uptake in AO and BO organoids over a range of concentrations (0-100 µg/mL) using fluorescence intensity and Phasor FLIM events counting approaches. AO and BO organoid data from each concentration group were compared with Mann-Whitney test (at p value 0.05). Data represents replicate R1 of 2 independent repeats.

Intensity analysis									
[NP D], µg / mL	N1 (AO) N2 (BO)	U	Z	Exact prob>|U|	Asymp. Prob >|U|	Median, (AO/BO)	Mean Rank (AO/BO)	Sum Rank (AO/BO)	Organoid loading events, % (AO /BO)
noNP	N1 =23 N2 =13	150	0	1	1	15.774 / 16.434	18.5 / 18.5	426 /240	n/a
0.01	N1 =26 N2 = 17	236	0.3602	0.72161	0.71871	13.8 / 10.02	22.6/21.1	587/359	n/a
0.1	N1 =24 N2 = 10	28	-3.4584	2.12E-04	5.43E-04	27.259 / 60.5275	13.7 / 26.7	328 / 267	n/a
1	N1 =24 N2 = 27	99	-4.2366	<0.0001	<0.0001	9.904 / 59.698	16.6 / 34.3	399 / 927	n/a
10	N1 = 16 N2 = 12	42	-2.4837	1.13E-02	0.013	160.225/833.204	11.1/19	178 / 228	n/a
25	N1 =13 N2 = 18	14	-4.1033	<0.0001	<0.0001	22.106 / 998.5075	8.1 / 21.7	105 / 391	n/a
50	N1 = 18 N2 =16	78	-2.26	0.0224	0.02382	5.2535 / 1922.243	13.8 / 21.6	249 / 346	n/a
100	N1 = 13 N2 = 6	8	-2.675	0.00472	0.00747	352.788 / 7725.37	7.6 / 15.2	99 / 91	n/a
**Phasor FLIM event counting analysis**									

[NP D], µg / mL	N1 (AO) N2 (BO)	U	Z	Exact prob>|U|	Asymp. Prob >|U|	Median, (AO/BO)	Mean Rank (AO/BO)	Sum Rank (AO/BO)	Organoid loading events, % (AO /BO)
noNP	N1 =7 N2 =11	38.5	0	1	1	0/0	9.5 /9.5	66.5 /104.5	0/0
0.01	N1 =12 N2 = 12	35	-2.4228	0.01164	0.0154	0 / 3.9814E-5	9.4/15.6	113/187	17.7 /58.3
0.1	N1 = 9 N2 = 9	0	-3.5505	<0.0001	3.85E-04	4.04392E-5 / 7.12172E-4	5//14	45/126	55.6 /100
1	N1 = 5 N2 = 7	0	-2.8104	2.53E-03	4.95E-03	0 / 7.31422E-4	3//9	15/63	20 /100
10	N1 = 13 N2 =4	0	-2.8873	8.40E-04	0.00389	0.00379 /0.03488	7/15.5	91/62	92.3 / 100
25	N1 = 6 N2 = 8	14	-1.2264	0.22844	0.22003	0.00965 / 0.03202	5.8 /8.8	35 /70	83.3 / 100
50	N1 = 5 N2 = 9	2	-2.6667	0.004	0.00766	0.01991 / 0.09227	3.4 /9.8	17/88	100 /100
100	N1 = 5 N2 = 8	7	-1.8298	0.06527	0.06728	0.07511 /0.23885	4.4/8.6	22/69	100 /100

Table S5. Comparison of NP D uptake in AO organoids. Data represents one of two individual experimental replicates (R2), shown in Fig. 4C,D.

**Intensity-based analysis (AO organoids)**							
Kruskal-Wallis ANOVA				Conover's post-hoc comparison to no NP group			
[NP D], µg / mL	AO median	Mean Rank	Sum Rank	Mean Rank Difference	Z	Prob	Difference at p=0.05
noNP	52.1	22.2	333	0	n/a	n/a	n/a

0.01	77.8	41.6	790	-19.37895	-2.26213	0.20184	n.s.
0.1	91.5	53.3	856	-31.3	-3.51134	0.006	s.d.
1	59.7	36.1	613	-13.85882	-1.57734	0.4839	n.s.
10	186.5	87.3	2182	-65.08	-8.0341	7.03E-12	s.d.
25	289.4	99.3	2283	-77.06087	-9.36173	4.29E-15	s.d.
50	501	110.6	1770	-88.425	-9.91983	1.70E-16	s.d.
100	1653.3	129.4	2199	-107.15294	-12.1956	2.57E-22	s.d.
Test statistics (at p level 0.05): Chi-Square 100.13455, DF 7, Prob > Chi-Square is < 0.0001							
**Phasor FLIM event counting analysis (AO organoids)**							
Kruskal-Wallis ANOVA				Conover's post-hoc test comparison to no NP group			
[NPD], µg / mL	AO median	Mean Rank	Sum Rank	Mean Rank Difference	Z	Prob	Difference at p=0.05
noNP	0	30	390	0	n/a	n/a	n/a
0.01	0	31.5	661	-1.47619	-0.25882	1	n.s.
0.1	0	43.8	569	-13.76923	-2.17213	0.25677	n.s.
1	0	35.6	498	-5.57143	-0.89503	1	n.s.
10	2.44E-03	65.3	653	-35.3	-5.1928	1.43E-05	s.d.
25	2.07E-03	77.5	1317	-47.47059	-7.97221	4.23E-11	s.d.
50	2.06E-03	84.4	844	-54.4	-8.0025	3.80E-11	s.d.

100	1.11E-02	100.6	1509	-70.6	-11.5282	6.26E-19	s.d.
Test statistics (at p level 0.05): Chi-Square 82.1359, DF 7, Prob > Chi-Square is < 0.0001							

Table S6. Comparison of NP D uptake in BO organoids. Data represents 1 of 2 individual experimental replicates (R2), shown in Fig. 4C,D.

**Intensity-based analysis (BO organoids)**							
Kruskal-Wallis ANOVA				Conover's post-hoc comparison to no NP group			
[NP D], µg / mL	BO median	Mean Rank	Sum Rank	Mean Rank Difference	Z	Prob	Difference at p=0.05
noNP	64.7	12.5	162	0	n/a	n/a	n/a
0.01	91.4	24.8	323	-12.3846	-2.16388	0.09713	n.s
0.1	177.9	47.4	1043	-34.9476	-6.84635	3.04E-09	s.d.
1	155.7	39.8	676	-27.3032	-5.07857	9.42E-06	s.d.
10	1791.7	81.4	1384	-68.9502	-12.8252	2.86E-23	s.d.
25	2462.8	85.9	1633	-73.4858	-13.9917	5.01E-26	s.d.
50	5484.7	109.9	2198	-97.4385	-18.7436	1.09E-36	s.d.
100	12039.2	124.3	1492	-111.872	-19.1516	1.52E-37	s.d.
Test statistics (at p level 0.05): Chi-Square 114.07957, DF 7, Prob > Chi-Square is < 0.0001							
**Phasor FLIM event counting analysis (BO organoids)**							
Kruskal-Wallis ANOVA				Conover's post-hoc test comparison to no NP group			

[NP D], µg / mL	BO median	Mean Rank	Sum Rank	Mean Rank Difference	Z	Prob	Difference at p=0.05
noNP	0	7.5	67.5	0	n/a	n/a	n/a
0.01	2.14E-05	12.4	123.5	-4.85	-1.36381	0.53524	n.s
0.1	5.82E-04	31.3	532	-23.7941	-7.45755	1.36E-09	s.d.
1	1.20E-03	36.1	325	-28.6111	-7.84167	2.78E-10	s.d.
10	2.51E-02	58.6	410	-51.0714	-13.0935	4.63E-20	s.d.
25	1.11E-02	54.5	654	-47	-13.7711	3.21E-21	s.d.
50	8.34E-02	71.5	787	-64.0455	-18.4102	1.05E-28	s.d.
100	0.16887	76.5	842	-69.0455	-19.8475	8.92E-31	s.d.
Test statistics (at p level 0.05): Chi-Square 77.47353, DF 7, Prob > Chi-Square is < 0.0001							

Table S7. Comparison of NP D uptake in AO organoids (R1 independent replicate).

**Intensity-based analysis (AO organoids)**							
Kruskal-Wallis ANOVA				Conover's post-hoc comparison to no NP group			
[NP D], µg / mL	AO median	Mean Rank	Sum Rank	Mean Rank Difference	Z	Prob	Difference at p=0.05
0	15.774	67.4	1550	0	n/a	n/a	n/a
0.01	13.802	70.7	1837	-3.26254	-0.26517	1	n.s.
0.1	27.259	75.8	1818	-8.3587	-0.66645	1	n.s.
1	9.904	61.3	1471	6.09964	0.48633	1	n.s.
10	160.225	109.4	1751	-42.0462	-3.00488	7.48E-02	n.s.
25	22.106	82.6	1074	-15.2241	-1.02076	1	n.s.
50	5.2535	76.3	1373	-8.88647	-0.65697	1	n.s.
100	352.788	117.6	1529	-50.2241	-3.36748	2.51E-02	s.d.

Test statistics (at p level 0.05): Chi-Square 22.8339, DF 7, Prob > Chi-Square is 0.00182							
**Phasor FLIM event counting analysis (AO organoids)**							
Kruskal-Wallis ANOVA				Conover's post-hoc test comparison to no NP group			
[NP D], µg / mL	AO median	Mean Rank	Sum Rank	Mean Rank Difference	Z	Prob	Difference at p=0.05
0	0	14	98	0	n/a	n/a	n/a
0.01	0	16.4	197	-2.41667	-0.55608	1	n.s.
0.1	4.E-05	24.4	220	-10.4444	-2.26805	0.27349	n.s.
1	0	17.6	88	-3.6	-0.67283	1	n.s.
10	3.79E-03	43.1	560	-29.0769	-6.78752	2.10E-07	s.d.
25	9.65E-03	42.3	254	-28.3333	-5.57324	1.47E-05	s.d.
50	1.99E-02	50.6	253	-36.6	-6.8404	1.80E-07	s.d.
100	7.51E-02	56.6	283	-42.6	-7.96178	3.07E-09	s.d.
Test statistics (at p level 0.05): Chi-Square 45.90195, DF 7, Prob > Chi-Square is < 0.0001							

Table S8. Comparison of NP D uptake in BO organoids (R1 independent replicate).

**Intensity-based analysis (BO organoids)**							
Kruskal-Wallis ANOVA				Conover's post-hoc comparison to no NP group			
[NP D], µg / mL	BO median	Mean Rank	Sum Rank	Mean Rank Difference	Z	Prob	Difference at p=0.05
0	16.434	20.2	262	0	n/a	n/a	n/a
0.01	10.024	23	391	-2.84615	-0.4573	0.64835	s.d.
0.1	60.5275	54.4	544	-34.2462	-4.81974	6.43E-05	s.d.
1	59.698	43.9	1186	-23.7721	-4.16864	6.70E-04	s.d.
10	833.204	79.5	954	-59.3462	-8.77584	4.40E-13	s.d.
25	998.5075	85.8	1545	-65.6795	-10.6822	2.20E-17	s.d.
50	1922.243	98.7	1577	-78.4087	-12.4309	2.52E-21	s.d.
100	7725.37	113.5	681	-93.3462	-11.1962	1.56E-18	s.d.
Test statistics (at p level 0.05): Chi-Square 91.38245, DF 7, Prob > Chi-Square is < 0.0001							
**Phasor FLIM event counting analysis (BO organoids)**							
Kruskal-Wallis ANOVA				Conover's post-hoc test comparison to no NP group			

[NP D], μg / mL	BO median	Mean Rank	Sum Rank	Mean Rank Difference	Z	Prob	Difference at p=0.05
0	0	8.5	93.5	0	n/a	n/a	n/a
0.01	3.98E-05	15.2	182.5	-6.70833	-3.63999	0.00228	s.d.
0.1	7.12E-04	30.7	276	-22.1667	-11.1703	4.39E-15	s.d.
1	7.31E-04	32.6	228	-24.0714	-11.2765	3.17E-15	s.d.
10	3.49E-02	47.3	189	-38.75	-15.032	1.09E-20	s.d.
25	3.20E-02	45.6	365	-37.125	-18.0965	1.33E-24	s.d.
50	9.23E-02	55.3	498	-46.8333	-23.6005	1.21E-30	s.d.
100	0.23885	64.3	514	-55.75	-27.1752	5.64E-34	s.d.
Test statistics (at p level 0.05): Chi-Square 63.96944, DF 7, Prob > Chi-Square is < 0.0001							

Table S9. Cluster point classification analysis of organoids loaded with a mixture of D and B NPs.

Organoid number	Polarity topology	Percentage of points in cluster per zone		
		G ≤ 0.5275 (zone D)	0.5275<G<0.6532 ('D+B' zone)	G ≥ 0.6532 (zone B)
B_D_1	BO	58.3048919	39.13538111	2.55972696
B_D_2_1	AO	23.2179226	71.28309572	5.49898167
B_D_3_1	AO	38.4615385	56.1965812	5.34188034
B_D_4_2	AO	18.8425303	32.03230148	49.1251682
B_D_7_1	AO	17.481203	58.92857143	23.5902256
B_D_7_2	AO	43.9655172	35.34482759	20.6896552
B_D_8	AO	21.2536729	51.3222331	27.424094
B_D_9	AO	15.9645233	54.98891353	29.0465632
B_D_10	AO	43.902439	39.9113082	16.1862528
B_D_11_1	AO	23.2502966	67.25978648	9.48991696
B_D_11_2	AO	37.4620829	46.86552073	15.6723964
B_D_11_3	AO	8.96551724	88.27586207	2.75862069
B_D_12_1	AO	72.8323699	24.27745665	2.89017341
B_D_12_2	AO	10.3862661	66.26609442	23.3476395
B_D_12_3	AO	22.7777778	54.72222222	22.5
B_D_13	AO	16.4961637	30.94629156	52.5575448
B_D_14_1	AO	45.2599388	44.03669725	10.7033639
B_D_14_2	AO	2.42460083	16.14429332	81.4311059
B_D_14_3	AO	31.1151079	62.41007194	6.47482014
B_D_15	BO	17.2165339	34.09177095	48.6916951
B_D_16	AO	20.1932367	55.24154589	24.5652174
B_D_17	BO	17.4045802	60.22900763	22.3664122
B_D_19	BO	30.5496829	34.03805497	35.4122622

B_D_20	ABO	35.2546917	35.79088472	28.9544236
B_D_21	ABO	34.7314202	55.99705666	9.27152318
B_D_22	BO	75.3571429	11.42857143	13.2142857
B_D_23	BO	51.6393443	28.41530055	19.9453552
B_D_24	BO	54.0669856	24.64114833	21.291866

Table S10. List of intestinal organoid lines used in the work.

Name	Species	Sex	Age	Date of primary culture isolation	Special features	Experiment type
mSIO Lgr5GFP	Mus musculus	female	N/A	2009	Lgr5-GFP labeled stem cells, from duodenum + jejunum	NP uptake test
porgj_2	Sus scrofa domesticus	female	7 weeks	11/24/2022	small intestinal organoids from pig jejunum	NP uptake test; ATP test, mitochondria analysis; chemokine expression
porgj_3	Sus scrofa domesticus	female	6-7 weeks	06/_/2024	small intestinal organoids from pig jejunum	NP uptake test; MNP co-loading test; phasor event method validation; chemokine expression
porgj_4	Sus scrofa domesticus	female	6-7 weeks	06/_/2024	small intestinal organoids from pig jejunum	NP uptake test
PigD	Sus scrofa domesticus	female	3 weeks	6/1/2024	small intestinal organoids from pig jejunum	chemokine expression
Pig13	Sus scrofa domesticus	male	5 weeks	7/1/2024	small intestinal organoids from pig jejunum	chemokine expression
Pig24	Sus scrofa domesticus	female	6 weeks	7/1/2024	small intestinal organoids from pig jejunum	chemokine expression

Supplementary videos (uploaded separately)

Supplementary Video S1: Time-lapse fluorescence intensity microscopy (1.5 min) showing NP B (magenta) co-localization with lysosomes (LysoSensor Green, cyan) in organoids. Scale bar: 10 µm. Organoid was imaged live with 1048x1048 resolution (76.34 x 76.34 µm physical size, pixel size 0.073 x 0.073 µm), 1A.U., 400 Hz scanning speed (pixel dwell time 1.363 µs), time interval 2.625s, 1 frame repetition.

Supplementary Video S2: Time-lapse fluorescence intensity microscopy (1.5 min) showing NP D (magenta) co-localization with lysosomes (LysoSensor Green, cyan) in organoids. Scale bar: 10 µm. Organoid was imaged alive with 1048x1048 resolution (76.34 x 76.34 µm physical size, pixel size 0.073 x 0.073 µm), 1A.U., 400 Hz scanning speed (pixel dwell time 1.363 µs), time interval 2.625s,1 frame repetition.

Supplementary Video S3: Time-lapse imaging (2 min) of NP D-exposed basal-out organoid (BO) after 3 days of treatment, labeled with WGA (green), TMRM (yellow), and NP D (magenta). Organoid was imaged alive with 1024x1024 resolution (61.21 x 61.21 µm physical size, pixel size 0.06 x 0.06 µm), 1A.U., 400 Hz scanning speed (pixel dwell time 1.038 µs), time interval 2.59s,1 frame repetition.

Supplementary Video S4: Time-lapse imaging (2 min) of control basal-out organoid (BO) after 3 days without NPD treatment, labeled with WGA (green) and TMRM (yellow). Organoid was imaged alive with 1024x1024 resolution (75.84 x 75.84 µm physical size, pixel size 0.07 x 0.07 µm), 1A.U., 400 Hz scanning speed (pixel dwell time 1.038 µs), time interval 2.59s,1 frame repetition.

Supplementary Video S5: Instruction for G, S coordinate extraction from raw .PTU files using custom Python code based on napari-flim-phasor-plotter plugin.

Supplementary Video S6: Time-lapse TMRM-FLIM (2min) showing mitochondrial dynamics in organoids. Organoid was imaged alive with 1024x1024 resolution (98.31 x 98.31 µm physical size, pixel size 0.07x 0.07 µm), 1A.U., 400 Hz scanning speed (pixel dwell time 1.038 µs), time interval 2.59s,1 frame repetition. FLIM data displayed with lifetime range 0-4 ns. Scale bar: 10 µm.

Supplementary methods

CXCL8 ELISA. CXCL8 was quantified in the culture supernatant of apical-out enteroids using a swine-specific CXCL8 DuoSet ELISA kit (R&D systems, Minneapolis, MN, USA) following the manufacturer's instructions. Culture supernatant was diluted ½ in reagent diluent. Absorbance values (450 nm) were measured using a Tecan Spark and converted to concentrations using Deltasoft software. The CXCL8 concentrations were then normalized to the protein concentration of apical-out enteroid lysates for each condition. Protein concentrations were determined using BCA.

Total ATP measurements in organoids. This was performed using a CellTiter-Glo viability assay (Promega, G7591) with following modifications. Two independent experimental repeats were done on the same organoid line at different passages. Four domes of BO porgj-2 organoids were used to produce 4100 µL of AO organoid suspension in HGM. The suspension was dispensed at 200 µL per well in 20 wells of a flat bottom 96 well plate (Greiner) pre-treated with

Lipidure™ coating solution. Nanoparticles type D were immediately added to 10 wells in a final concentration 10 µg / mL, leaving untreated 10 control wells and incubated for 48 h, followed by replacement of HGM with imaging media (IM) without glucose with sequential (6 times) partial medium exchange, resulting in final 200 µL of medium per well (this procedure allowed to decrease glucose in the media to activate OxPhos in organoids). Subsequently, organoids were incubated for 24 h prior to ATP measurement on day 4. Nanoparticle loading efficiency (the percentage of nanoparticle-positive organoids from total number of organoids analyzed, with partially loaded organoids counted as one loading event) was assessed on a widefield inverted fluorescence microscope IX81, 40x/0.6 LUCPlanFLN objective (Olympus). For total ATP measurement in organoids at rest, first 130 µL of organoid suspension were treated with mock (DMSO) or 1.4 µM FCCP/ 7 µM oligomycin mixture for (20 min, 37 °C, 5% CO_2). Collectively, four different groups with 5 wells per group were tested: control / resting, control / stress, nanoparticles D treated / resting, nanoparticles D treated / stress. After treatment, suspensions from individual wells (140 µL) were collected into individual 500 µL lipidure-coated vials. To ensure complete collection of organoids, wells were additionally rinsed with 150 µL of no glucose IM, which was combined in the corresponding vials with the main organoid suspension. Organoids were collected by centrifugation (5 min, 300 g, RT), and the pellet was gently resuspended in 10 µL of media. Subsequently, 100 µL of ATP reagent (1:1 mixture of no glucose IM Cell Titer-Glo reagent) was added to individual vial and vigorously mixed (vortex) for 18 s. Vials were centrifuged (5 min, 300 g, RT) and 80 µL of debris-free supernatant was collected into white opaque-walled 96 well plate (Corning), proceeded by the measurement of luminescence with Varioscan microplate-reader (Thermo Fisher Scientific) with 1000 ms exposure time. The background noise signal was determined as an average signal measured from 5 individual wells with 80 µL of media taken from the mixture of 10 µL of no glucose IM with ATP reagent. This was subtracted from a total luminescence signal of each individual experimental well and normalized per total average area square of organoid per treatment condition (measured by transmission light microscopy of individual sample wells of 96 well plate with widefield inverted microscope Olympus IX81).

Statistical analysis was done in OriginPro 12 (OriginLab) using Mann-Whitney non-parametric test to compare between groups of no NP (control) and NP D (loaded organoids with confirmed uptake) at resting (no stimulation) and FCCP / Oligomycin stimulation conditions. The n for each group was 5 wells with organoid suspensions.

Phasor plot analysis of MNP effects on mitochondrial polarization. To study the effect of NP on mitochondrial polarization in intestinal organoids, we exported TMRM FLIM data in PTU format from LAS X software and processed them using the napari-flim-phasor-plotter plugin (https://zenodo.org/records/12620956) and ClaudeAI designed macros code 'Supplementary Code 1' (https://github.com/HangZhouFLIM/FLIM_nano). For each PTU file from control (no NP) and NP D groups (days 1 and 3), we selected the TMRM channel and manually cropped the organoid region to remove artifacts. Phasor analysis was performed (bin size=1, threshold=10, median filter=10) at 80 MHz laser frequency. G-S coordinates and ROI measurements were exported as .csv files. We then calculated the mean G and S from G and S coordinates, with data filtering (2.5-97.5 percentile outlier removal of G and S respectively) to remove extreme values, and converted to mean $\tau_{(m)}$ using the equation: $\tau\_m = 1/\omega\ \sqrt{((1 - G^2 - S^2)/(G^2 + S^2\ ))}$ with ω = 80 MHz and draw the boxplot in control (no NP) and NP D groups (days 1 and 3) using ClaudAI designed 'Supplementary Code 2' (https://github.com/HangZhouFLIM/FLIM_nano). The statistical analysis was done with Mann-Whitney test at the significance level $p < 0.05$ by application of the 'Supplementary Code 2'.

Mitochondrial dynamics and morphology analysis. To investigate impact on the mitochondrial dynamics and morphology, AO organoids (Porgj-2) were cultured for 24 h before NP D treatment, followed by additional 1- or 3-day cultures. At each timepoint, organoids were stained with WGA (1 h), washed three times with imaging medium and then treated with TMRM to visualize mitochondria. Live microscopy was performed using a Stellaris Falcon 8 confocal microscope (1024 × 1024 pixels, 400 Hz scan speed, excitation: 510 nm, emission: 555-650 nm) with 2-minute time-lapse acquisition yielding 47 sequential timeframes (t1 to t47). Images were exported as OME-TIFF format using LAS X software and preprocessed to separate WGA, TMRM, and NP D channels using custom Python scripts in napari ('Supplementary Code 3', https://github.com/HangZhouFLIM/FLIM_nano). Mitochondrial features (TMRM images) were tested using the Nellie plugin in napari (https://zenodo.org/records/13863809) for single timeframes, and multiple timeframes were processed through custom batch-processing scripts based on Nellie code from GitHub ('Supplementary Code 4', https://github.com/HangZhouFLIM/FLIM_nano) and exported as CSV files. Ten key parameters were selected: linear velocity, angular velocity, tortuosity, aspect ratio, length, solidity, extent, minor axis length, major axis length, and area, and data analysis was performed at organoid level (mean values of all mitochondria per organoid, visualized with box plots) comparing NP and no NP groups on day 1 and day 3 using custom analysis scripts ('Supplementary Code 5', https://github.com/HangZhouFLIM/FLIM_nano). The statistical analysis was done with Mann-Whitney test at the significance level $p<0.05$ by application of the 'Supplementary Code 5'.
